# Phylogenetic implications of nuclear rRNA IGS variation in *Stipa* L. (Poaceae)

**DOI:** 10.1038/s41598-017-11804-x

**Published:** 2017-09-14

**Authors:** Katarzyna Krawczyk, Marcin Nobis, Arkadiusz Nowak, Monika Szczecińska, Jakub Sawicki

**Affiliations:** 1 Department of Botany and Nature Protection, Faculty of Biology and Biotechnology, University of Warmia and Mazury in Olsztyn, Olsztyn, Poland; 2 Institute of Botany, Faculty of Biology and Earth Sciences, Jagiellonian University, Kraków, Poland; 3 Polish Academy of Sciences Botanical Garden – Center for Biological Diversity Conservation in Powsin, Warsaw, Poland

## Abstract

The article takes up the problem of deficiency of molecular marker, which could illustrate molecular variability as well as phylogenetic relation within the genus of *Stipa* L. (Poaceae). Researches made so far hadn’t delivered sufficient information about relationships between particular taxa from the genus of *Stipa*. In the present study, we analyzed variability and phylogenetic informativeness of nuclear ribosomal DNA in six species from the genus against five other species from Poaceae including a division of this region into functional elements and domains. Our results showed that the intergenic spacer region, and especially its part adjacent to 26 S nrDNA, is a molecular marker giving a real chance for a phylogeny reconstruction of *Stipa*. The region seems to be the most phylogenetically informative for *Stipa* from all the chloroplast and nuclear markers tested so far. Comparative analysis of nrDNA repeat units from *Stipa* to other representatives of Poaceae showed that their structure does not deviate from the general scheme. However, the rate of evolution within the inter-repeats in the IGS region is extremely high and therefore it predestines the region for phylogenetic analyses of *Stipa* at genus level or in shallower taxonomic scale.

## Introduction

The tribe Stipeae comprised over 580 species, common or dominant in open grassland and steppes, with species diversity in temperate regions of Asia, Europe, Africa, Australia and America^1^. Currently, based on morphological and molecular data, the species belonging to the tribe were segregated into several genera^2–5^, with *Stipa* L., *Nasella* (Trin.) E.Desf., *Achnatherum* P.Beauv., *Austrostipa* S.W.L.Jacobs & Everett, *Jarava* Ruiz & Pav., *Piptatherum* P.Beauv. and *Piptochaetium* J.Presl. being the richest in species^4^. Phylogenetic studies, based on cpDNA and ITS data have shown, that some of genera within Stipeae like, *Jarava*, *Achnatherum*, *Piptochaetium*, *Austrostipa*, *Celtica* F.M.Vazquez & Barkworth, *Patis* Ohwi, *Hesperostipa* (M.K.Elias) Barkworth, constituted well resolved and highly credible clades^1, 3–7^. However there is still some groups e.g. within *Ptilagrostis* Griseb, *Piptatherum* P.Beauv., *Nasella*, *Achnatcherum* and especially in *Stipa* s. str., in which phylogenetic relationships remined unresolved^3–5^. With over 160 species (Nobis npbl.) native to Asia, Europe and north Africa, *Stipa* s. str. is currently the richest in species genus within Stipeae. However, in the light of existing researches using molecular method, only small group of Himalayan species comprised *Stipa capillacea* Keng, *S*. *regeliana* Hack., *S*. *purpurea* Griseb., *S*. *penicillata* Hand.-Mazz. and *S*. *roborowskyi* Roshev. are separated from remaining *Stipa* species representing almost all distinguished sections within the genus^3, 4^. There is distinctly a lack of molecular method, that could explain both, molecular variability between selected taxa as well as phylogenetic relation within the genus *Stipa*. Thus, the aim of our paper was to find a molecular marker representing a sufficient level of informativeness to play a role of phylogenetic marker for *Stipa*.

In our research, we focused on the repeat units of nuclear RNA genes (nrDNA). In higher plants nrDNA is usually organized in long tandem repeats forming within the chromosomes the nucleolar organizing regions (NOR)^8^. The nrDNA repeat units comprise DNA coding for RNA subunits and non-coding regions, which are widely applied in plant phylogenetics^9–11^. Moreover, the region provides great potential to study historical or recent hybridization as well as introgression events. It is because of the fact that in case of reticulate evolution individual nrDNA copies are not homogenized immediately. For this reason, multiple divergent rDNA copies, representing different ribotypes, originating from orthologues and paralogues, can be simultaneously present in a nuclear genome^11–13^. Phylogenetic marker of such properties would be especially useful in studies on *Stipa*, since hybrid origin of some species representing the genus have been reported^14–17^.

The present study is the first report on complete sequence of nrDNA in *Stipa* and in the tribe Stipeae. The analysis of nrDNA structure in *Stipa* spp. was carried out in comparison with nrDNA sequences of *Brachypodium distachyon* (L.) P.Beauv., *Oryza sativa* L., *Setaria italica* (L.) P.Beauv., *Sorghum bicolor* (L.) Moench and *Zea mays* L., which constitute all the available data on complete nrDNA units published for the Poaceae family.

## Results

### Structural organization of nrDNA

**Figure 1:**
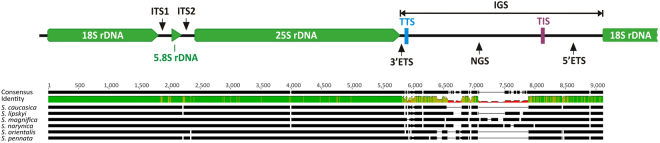
nrDNA unit in *Stipa*. Structural organization scheme of the nulear-encoded nrDNA repeat unit in *Stipa*.

In the examined species nrDNA repeat units followed a common organizational scheme (Fig. 1). A single unite comprised genes coding for 18 S, 5.8 S and 25 S nrRNA and three non-coding regions: Internal Transcribed Spacer 1 (ITS1), Internal Transcribed Spacer 2 (ITS2) and Intergenic Spacer (IGS). An exception to this scheme was *Oryza sativa*, where due to the order of functional elements and an occurrence of only one nrDNA unit in the analyzed chromosome, ITS2 could not be distinguished.

The analyzed IGS regions consisted of three functional elements: 3′ External Transcribed Spacer (3′ETS), Non-Transcribed Spacer) NTS and 5′ External Transcribed Spacer 5′ETS (Fig. 1). 3′ETS is a short nucleotide sequence lying downstream 3′ end of 25 S nrRNA gene and ending with transcription termination site (TTS). The exact location of pyrimidine reach motif characteristic for TTS is presented in Supplementary Table S1. In IGS of *Stipa* we found one putative TTS, while in each of the other species studied two putative TTSs were detected. On the other end of IGS the 5′ETS region is located. Its beginning is determined by a transcription initiation site (TIS) while its end is placed upstream 5′ end of 18 S nrDNA. In the analyzed sequences, the number of putative TIS ranged from one in *Zea mays* to four in *Brachypodium distachyon* (Supplementary Table S2). Between 3′ETS and 5′ETS a non-transcribed spacer (NTS) was located demonstrating high variability in sequence length, since only within one genus (*Stipa*) NTS length ranged from 1453 bp (*S*. *caucasica*) to 2347 bp (*S*. *magnifica*).

### Variability of rDNA in *Stipa*

Sequence analysis comprising six species of the genus *Stipa* revealed a low level of genetic variation in the ITS1 and ITS2 regions (Table 1). Pairwise identity within the analyzed set of sequences amounted to 98.2% in ITS1 and 93% in ITS2. The contribution of variable (*V*) and parsimony-informative (*Pi*) sites in ITS1 reached only 4.52% and 1.36% respectively. Within the ITS2 seven variable characters (*V* = 3.38%) were found including only one parsimony informative site (*Pi* = 0.48%). This indicates that ITS2 was even more conservative than a complete rDNA unit. Compared to both ITS regions, IGS was characterized by a significantly higher level of genetic variation with *V* = 9.99%, *Pi* = 3.14% and 6% of singleton sites (*S*). However, the variation within the IGS was not homogenous, as evidenced by comparing 5′ETS with 3′ETS + NTS. Whilst the region 5′ETS was more conservative, at a similar level as was detected in the ITS1, 3′ETS analyzed together with NTS was the most variable fragment of the whole rDNA. For 3′ETS + NTS pairwise identity amounted to 65.9%, whereas the contribution of *Pi* sites reached 3.65% out of 11.49% of variable characters.

**Table 1:** Comparison of domains within rDNA. Characteristics of selected functional elements and domains within rDNA from *Stipa* spp. In the case of species where more than one putative TIS was found, the variant with the shortest 5′ETS was considered.

	rDNA	ITS1	ITS2	IGS	3′ETS + NTS	5′ETS
Alignment length, bp	9114	221	207	3314	2628	686
Sequence length, bp	7991–8897	221	205–207	2193–3098	1513–2414	677–684
Pairwise identity, %	91.0%	98.2%	93.0%	73.4%	65.9%	97.6%
Variable characters (V)	362 (3.97%)	10 (4.52%)	7 (3.38%)	331 (9.99%)	302 (11.49%)	29 (4.23%)
Parsimony-informative sites (Pi)	113 (1.24%)	3 (1.36%)	1 (0.48%)	104 (3.14%)	96 (3.65%)	8 (1.17%)
Singleton sites (S)	221 (2.42%)	7 (3.17%)	6 (2.90%)	199 (6%)	178 (6.77%)	21 (3.06%)

### Phylogenetic informativeness

**Figure 2:**
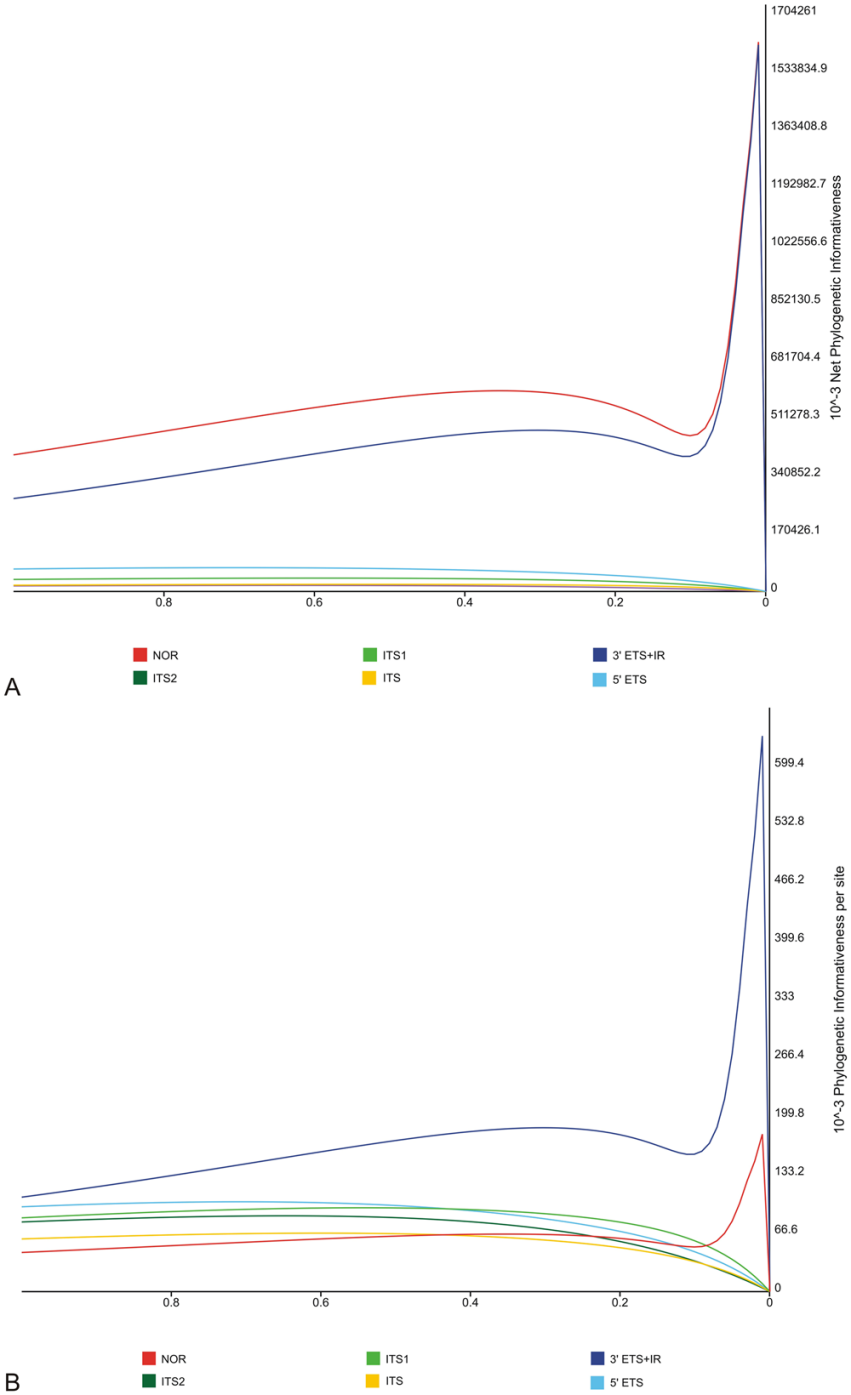
Phylogenetic informativeness. Profile of (**A**) net and (**B**) per site phylogenetic informativeness for selected regions within rDNA. The time axis was calibrated to the ultrametrisized ML tree calculated for the complete rDNA unit. An evolutionary time of 1.0 corresponds to the root of the tree and the value of 0 to its tips.

Functional elements and domains distinguished within the rDNA repeat unit were analyzed for the profile of phylogenetic informativeness (PI). A set of data comprising *Stipa* spp. and *Brachypodium distachyon* was analyzed separately for: the complete rDNA unit, ITS1, ITS2, the whole ITS region including 5.8 S rDNA, 5′ETS and for 3′ETS together with NTS. The analysis of net PI profiles, relating the overall informativeness of the nucleotide sequence, confirmed very low phylogenetic signal carried by ITS1 and ITS2 parsed both individually and together as the ITS (Fig. 2A). A low level of net PI was also detected in the 5′ETS region. The highest values of net PI were obtained for the complete rDNA unit and only slightly lower for the 3′ETS + NTS fragment. However, within the peak and on its right site PI profiles for rDNA and 3′ETS + NTS were overlapping (Fig. 2A). In turn, considering the length of the nucleotide sequence, significantly stronger phylogenetic signal was reported for 3′ETS + NTS (Fig. 2B). Thus, concerning both the results of net and per site PI, the most informative phylogenetic marker for *Stipa* was the fragment of IGS including 3′ETS and NTS.

**Figure 3:**
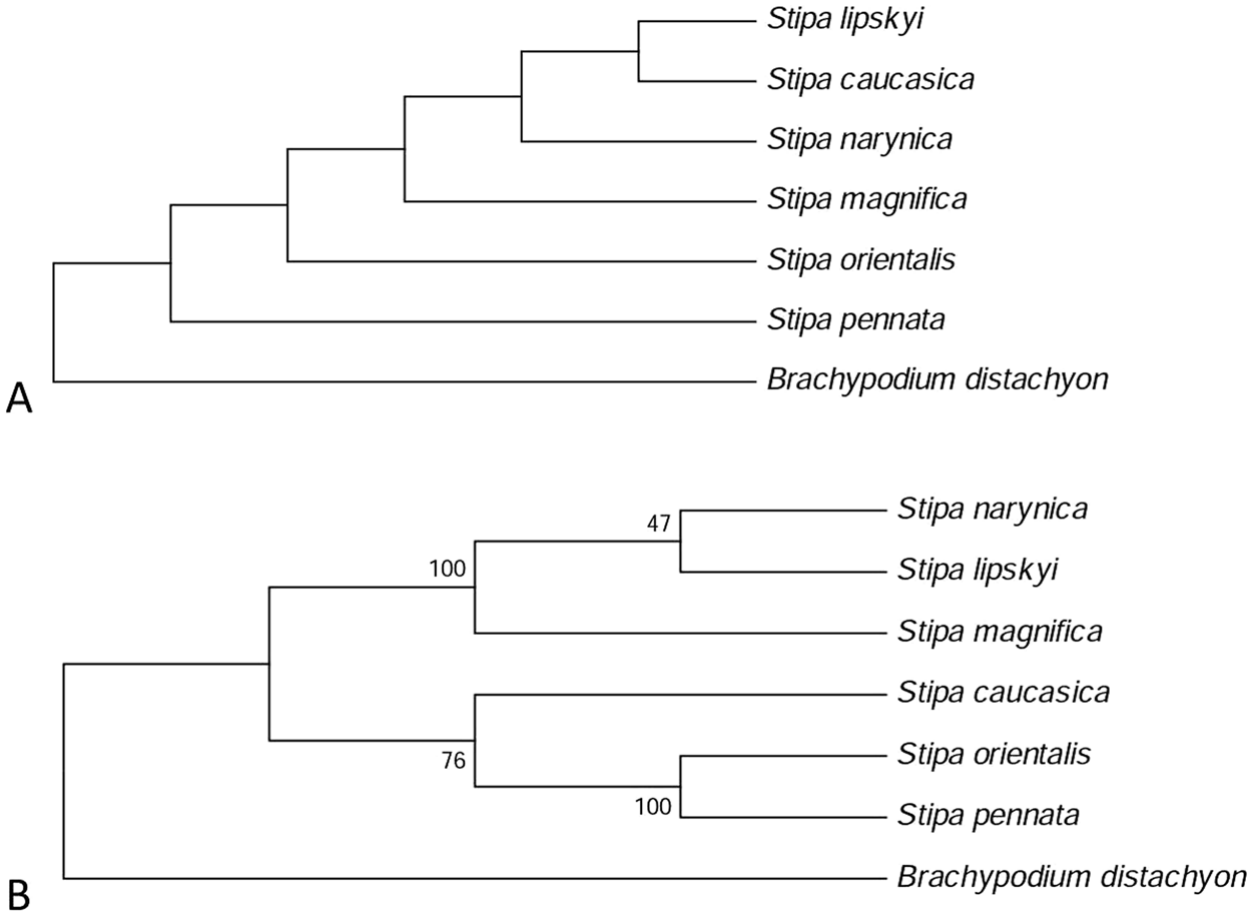
Comparison of ITS and 3′ETS + NTS resolving power. Consensus cladograms derived from MP analysis of (**A**) the complete ITS and (**B**) 3′ETS + NTS region. Bootstrap values lower than 60% are hidden.

The result was confirmed by the analysis of cladograms calculated separately for the complete ITS and for the 3′ETS + NTS region derived with Maximum Parsimony (MP) method (Fig. 3). The phylogenetic tree based on ITS sequence variation consists of weakly supported clades with all the bootstrap values below 60%. On the contrary, credibility of clades within the tree based on the 3′ETS + NTS region reached up to 100%. Only the clade comprising *Stipa lipskyi* and *S*. *narynica* was supported with 50% bootstrap. Furthermore, the compared cladograms significantly differed in respect of location of *S*. *caucasica*. The phylogenetic reconstruction derived from ITS indicated a close relationship of *S*. *caucasica* and *S*. *lipskyi* (Fig. 3A), while the analysis of sequence variation within 3′ETS + NTS placed these species into separate phylogenetic lines with a high degree of certainty (Fig. 3B).

**Figure 4:**
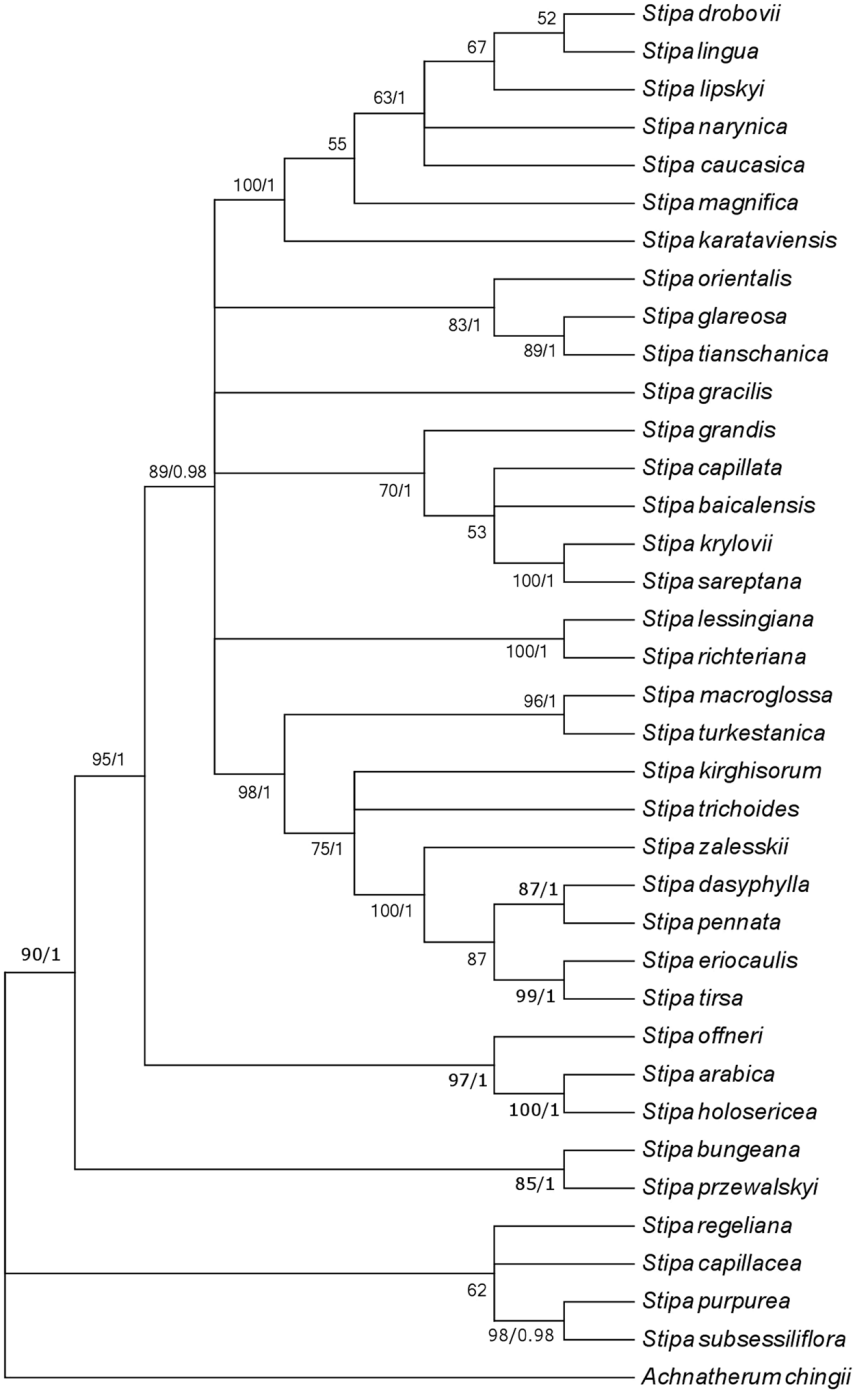
3′ETS + NTS -based cladogram. The 50% majority-rule bootstrap consensus tree inferred from 500 replicates derived from MP analysis of 3′ETS + NTS region. Bootstrap values >50% above, Bayesian of *P* > 0.95 below branches.

To verify applicability of 3′ETS + NTS as a phylogenetic marker for *Stipa* we performed the MP analysis on a set of sequences comprising 36 *Stipa* spp. (Supplementary Table S1) and *Achnatherum chingii* (Hitchc.) Keng as an outgroup. The length of analyzed sequences ranged from 314 bp in *Stipa bungeana* Trin. ex Bunge to 925 bp in *S*. *holosericea* Trin. The alignment had a total length of 1238 bp, contained 326 *Pi* sites and was characterized by a 67.5% sequence pairwise identity. The bootstrap consensus tree inferred from 500 replicates derived from MP analysis (Fig. 4) is well-resolved and fully consistent with the most parsimonious tree (1427 steps, consistency index = 0.559659) and with results of Bayesian inference (BI) analysis.

### Inter-repeat region

**Figure 5:**
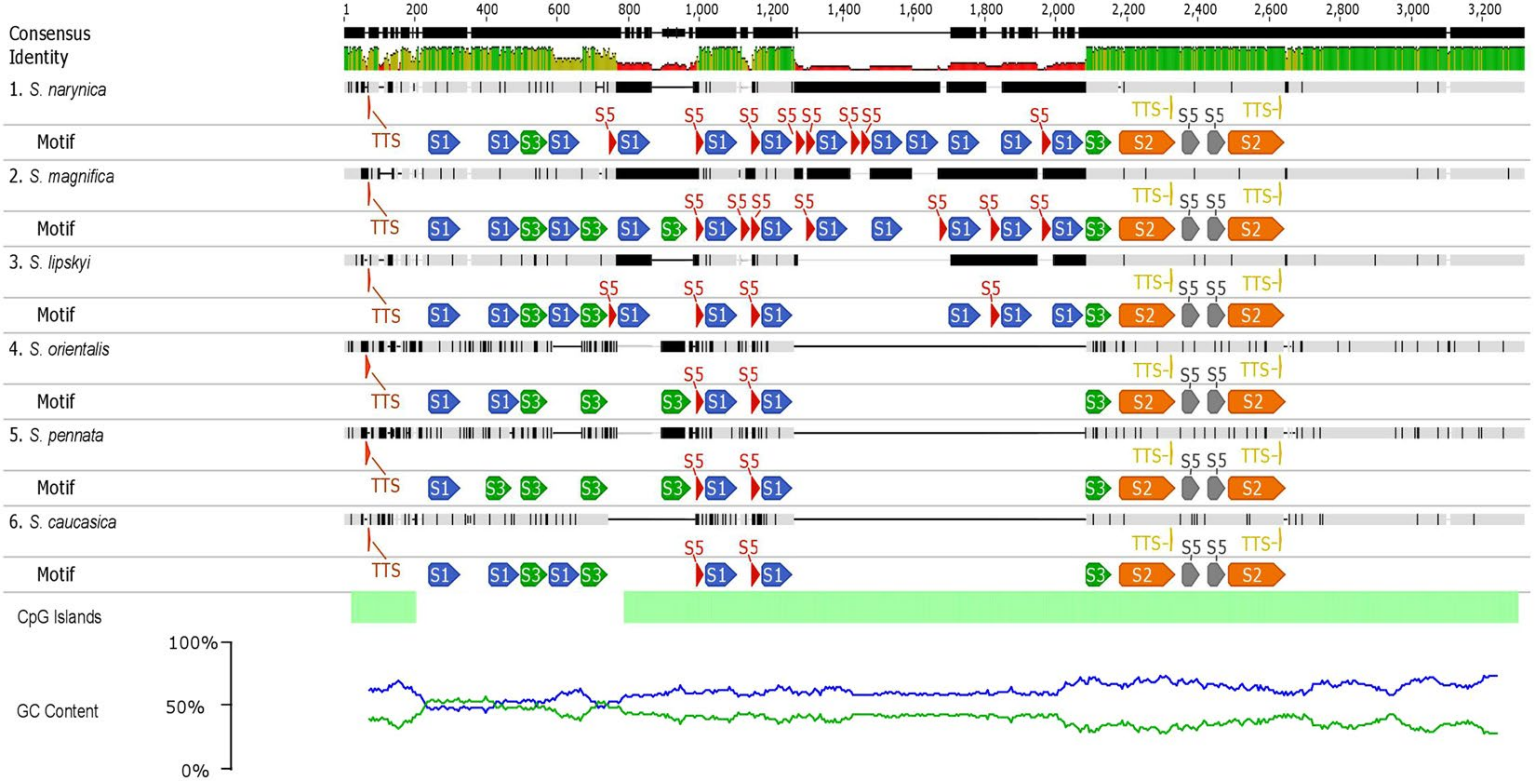
Structure of the IGS of nrRNA in *Stipa*. Blue line – plot of GC content, green line – plot of AT content; green bar – best global reconstruction of CpG islands. Colored bars – repeated regions.

For a detailed cognition of IGS variability, sequence self-comparison was carried out using dot-plot analysis (see Supplementary Figs S5–S15). The analysis revealed a higher level of sequence conservativeness in the beginning and the end of each IGS and a presence of inter-repeats (IR) region located between them. An identification of number and length of the repetitive sequence motifs occurring in *Stipa* allowed for defining five different IRs ranging from 28 to 162 bp (Table 2). There was a single nucleotide polymorphism (SNP) observed between particular IR copies within a species. The number of IR copies was highly variable between the species. For example, IR S1 (91 bp in length) was found in only three repeats in *S*. *pennata and S*. *caucasica* however in *S*. *narynica* it was present in 12 copies (Fig. 5.), what strongly affected the length of the entire IGS. Similarly, inter-repeat S5 occurred in two copies in *S*. *caucasica*, *S*. *orientalis* and *S*. *pennata* whilst eight repeats were found in *S*. *narynica*.

**Table 2:** Inter-repeats. Characteristics of repeated regions defined for the species analyzed in the study.

Species	Inter-repeat	E-value	Sites per species	Width, bp
*Stipa* spp.	S1	1.5e-738	3–12	91
	S2	6.4e-696	2	162
	S3	3.8e-636	2–5	77
	S4	8.4e-189	2	55
	S5	2.4e-168	2–8	28
*Brachypodium distachyon*	B1	8.7e-031	4	78
	B2	5.3e-016	5	39
	B3	3.2e-005	5	39
*Oryza sativa*	O1	7.8e-069	4	173
	O2	2.1e-012	5	57
*Setaria italica*	Si1	1.6e-057	5	77
	Si2	1.4e-008	5	25
*Sorghum bicolor*	Sb1	1.1e-075	3	212
	Sb2	2.2e-063	8	69
	Sb3	4.7e-051	5	69
	Sb4	2.1e-030	8	48
	Sb5	3.2e-012	5	25
*Zea mays*	Z1	8.7e-177	10	192

**Figure 6:**
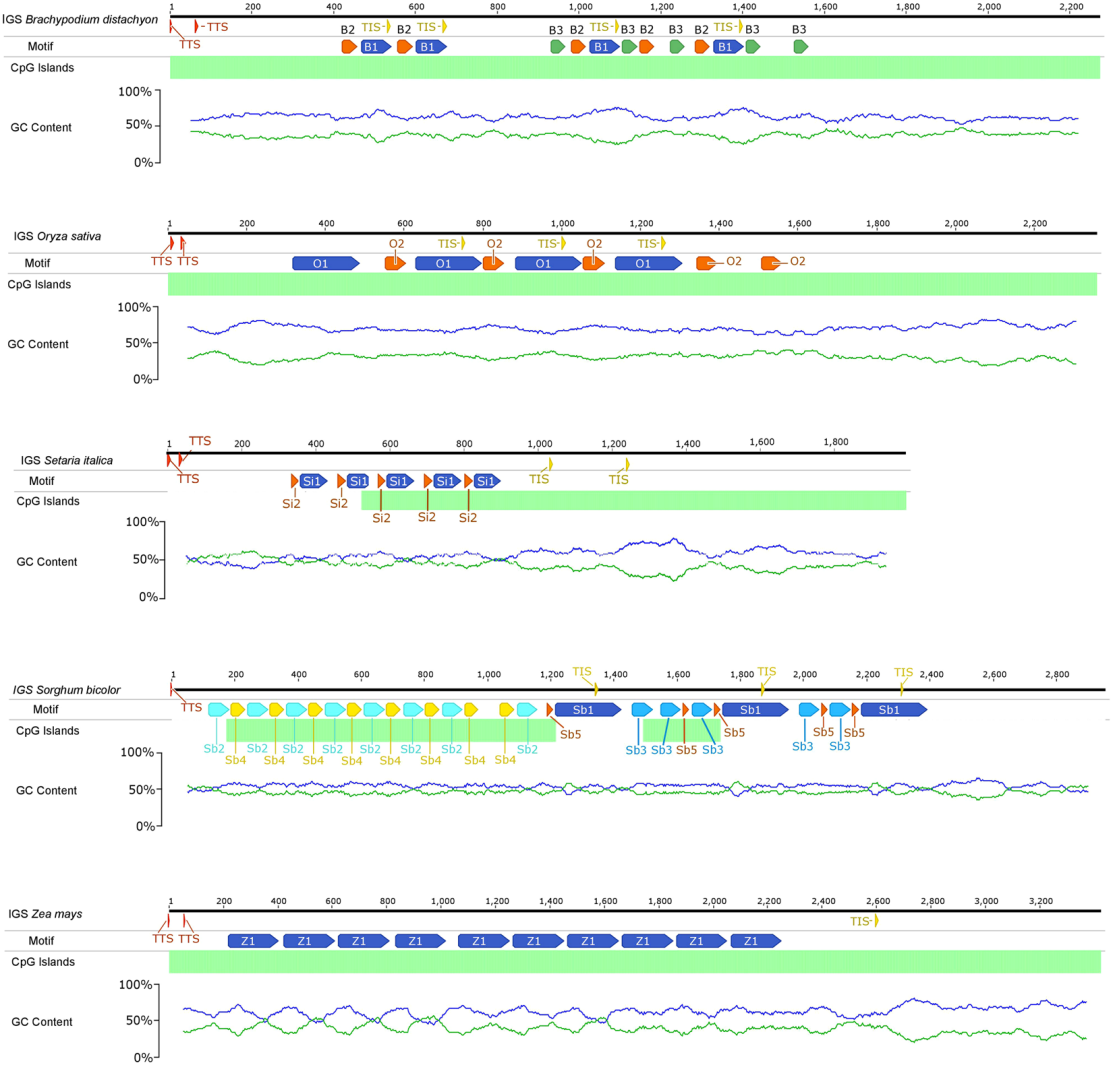
Structure of the IGS of nrRNA in Poaceae. Blue line – plot of GC content, green line – plot of AT content; green bar – best global reconstruction of CpG islands. Colored bars – repeated regions.

The comparison if IGS regions indicated an extremely high level of sequence diversity within the IR region. The simplest inter-repeat pattern was found in *Zea mays*, where only one type of IR occurred in 10 copies, each 192 bp in length. A simple scheme was also observed in IGS region from *Oryza sativa* where two nucleotide motifs were defined: O1 (173 bp in length) repeated four times and O2 (57 bp) present in five copies. In IGS from *Setaria italica* two motifs (Si1 = 77 bp, Si2 = 25 bp) present in five tandem repeats were found. In *Brachypodium distachyon* the analysis revealed the presence of three different inter-repeats ranging from 39 to 78 bp. The IR region in *Sorghum bicolor* could be distinguished into two parts. In a part located near the 25 S nrRNA gene motif Sb2 and Sb4 were alternated. Downstream there was located a second part of IR region comprising Sb1, Sb3 and Sb5 (Fig. 6). In all the species tested, excluding *S*. *italica* and *Z*. *mays* IRs were located both within NTS and 5′ETS. In nine of 11 examined species, repeated motifs included putative TIS, what entailed its multiplication from two in *Stipa* spp. to four in *B*. *distachyon* (Table 2).

### GC/AT-content and CpG islands

Within the rDNA, the highest share of GC-content reaching up to 76.3% (ITS2 of *Stipa magnifica*) was noted in ITS1 and ITS2 regions. The analyzed IGS were characterized by a lower contribution of GC pairs (Table 3), ranging from 53.6% in *Sorghum bicolor* to 70.1% in *Oryza sativa*. The distribution of GC pairs within the IGS region was variable between the analyzed species as well. In *Oryza sativa* and *Brachypodium distachyon* their even arrangement and relatively high share resulted in lack of AT–rich regions. In *S*. *bicolor* and *Zea mays* IGS contained four regions where the rate of AT pairs exceeded 50%. In the analyzed representatives of the genus *Stipa* a short AT-rich region was present within the NTS, near its 5′ end (Fig. 5). IGS from *Setaria italica* also contained a short region with a high rate of AT pairs within the 5′ part of NTS.

**Table 3:** Variability in length and GC-content. Comparison of selected functional elements and domains within the rDNA repeat unit between the analyzed species. GC – content of GC pairs in an analyzed nucleotide sequence. In the case of species where more than one putative TIS was found, the variant with the shortest 5′ETS was considered.

Species	rDNA		ITS1		ITS2		IGS		3′ETS + NTS		5′ETS	
	GC, %	Length, bp	GC, %	Length, bp	GC, %	Length, bp	GC, %	Length, bp	GC, %	Length, bp	GC, %	Length, bp
*Stipa caucasica*	58.5	7991	73.3	221	76.2	206	61.2	2193	59.5	1513	65.0	680
*S*. *lipskyi*	58.7	8451	73.8	221	75.7	206	61.1	2653	59.4	1969	65.9	684
*S*. *magnifica*	58.8	8897	72.9	221		207	61.2	3098	60.0	2414	65.6	684
*S*. *narynica*	58.7	8844	74.9	221	73.8	207	60.9	3045	59.5	2361	65.7	684
*S*. *orientalis*	58.4	8038	71.5	221	75.2	206	61.0	2239	59.5	1558	64.8	681
*S*. *pennata*	58.6	8031	71.0	221	75.1	205	61.7	2233	60.5	1556	64.4	677
*Brachypodium distachyon*	58.3	8078	63.5	222	69.0	216	62.9	2271	64.0	1392	61.1	879
*Oryza sativa*	55.0	7923	72.2	198	—	—	70.1	2357	69.6	1254	70.5	1103
*Setaria italica*	56.1	7779	57.4	209	61.5	213	57.3	1991	53.9	1212	62.6	779
*Sorghum bicolor*	54.3	8689	56.1	212	68.1	116	53.6	2952	53.5	2307	54.2	645
*Zea mays*	59.7	8795	69.9	219	74.1	216	62.5	3421	60.4	2591	69.2	830

The analysis of CpG sites location revealed they comprised the whole IGS from *B*. *distachyon*, *O*. *sativa* and *Z*. *mays*. In the IGS from *S*. *bicolor* there were two CpG-rich sites within the NTS (Fig. 6). In *Stipa* spp. the region characterized by a high concentration of CpG sites covered most of the IGS including 3′ETS, 5′ETS and a substantial part of the NTS. Only in the AT-rich fragment and downstream (Fig. 5) the lack of CpG islands was noted. In *Setaria italica* there was one CpG-reach region covering a part of NTS and the whole 5′ETS.

## Discussion

The parts of nuclear rDNA sequence data are widely used in phylogenetic inference at various taxonomic levels. The most prominent nuclear phylogenetic marker in plants is ITS^10^. The region was successfully applied for phylogeny reconstruction in Poaceae at the level of family^18^, subfamily^19^ and tribe^20, 21^. The ITS sequences were also shown to be useful for assessing evolutionary relationships among closely related grass species for example within the genera of *Bromus* ^22^, *Miscanthus* ^23^, *Hordeum* ^24^, *Elymus* ^25^, *Setaria* ^26^ and *Festuca-Lolium* complex^27^. However, the literature data have shown that phylogenetic trees derived from ITS sequences were unresolved within clades comprising *Stipa* ^3, 28^. The branch of ITS-based cladogram published by Hamasha *et al*.^3^ comprising *Stipa* spp. contained a lot politomies and only few clades with credibility value over 50%, which brought little information useful in reconstruction of phylogenetic relationships within the genus. Our study also revealed an insufficient level of ITS variability in the species from *Stipa* to address questions of their intrageneric relationships. Both the low-resolution phylogenetic tree derived from MP analysis and the profile of PI proved relatively low rate of ITS sequence evolution.

Much more information useful in phylogenetic assays concerning *Stipa* was carried by the IGS region. Numerous studies proved, that IGS was a valuable marker in phylogenetic analyses of angiosperms which could supplement nucleotide variation from generally shorter ITS^9, 11, 29^. The high rate of evolution of this spacer region makes it suitable even for detecting intraspecific polymorphism^8, 30–32^. The rate of evolution within IGS is not uniform, what is a rule for this region^12, 32^ and it presumably results from different functions of its components^8^. In general, more conservative region within IGS is the 5′ETS fragment^11, 12^. Literature data demonstrate that the variation of the fragment remains at level typical for ITS or slightly higher^12^. The same relation was demonstrated in *Stipa*, where the PI of IGS was greater than in ITSs, however the number of Pi sites did not exceed the number of Pi sites reported for ITS1.

A much higher level of informativeness identified in the IGS of *Stipa* was caused by a great variability of NTS containing repeated regions. Both the PI plot for 3′ETS + NTS and a cladogram, providing more resolution to phylogenetic reconstructions than the one obtained for ITS sequence, demonstrate that the phylogenetic signal carried by 3′ETS + NTS is strong enough to picture intrageneric relationships in *Stipa*. It was also confirmed by the MP and BI analysis conducted on a set of 36 *Stipa* species. 26 of 36 species included in our analysis were previously analyzed by Hamasha *et al*.^3^ and very low divergence of ITS sequence was reported for them. 3′ETS + NTS-based cladogram in comparison with ITS-based tree^3^ delivered much more information useful in phylogenetic inference in case of all the species common for the two studies. Considering only the species common to work by Hamasha *et al*.^3^ and our research, analysis based on ITS sequences resulted in only five clades at different levels of organization with bootstrap values over 50%, while the tree based on 3′ETS + NTS contained 19 clades with support >50%.

The part of intergenic spacer comprising 3′ETS + NTS is also more useful for phylogenetic implications in *Stipa* than cpDNA markers applied in the studies on this genus so far. For example, the phylogenetic analysis based on nine plastid DNA regions^4^ (Romaschenko *et al*. 2012) grouped *Stipa capillata*, *S*. *caucasica* and *S*. *pennata*, which represent three different sections within the genus, into one highly supported and unresolved clade. In turn, the analysis derived from the IGS variability placed these species into three different clades indicating their more distant relationship.

Unfortunately, the rapid evolution of NTS entails the presence of return mutations, homoplasies and substitution saturation, which make up a so-called informative noise^33^. Its presence is indicated by the PI profile with a peak placed near the right site of a diagram (Fig. 2). While the part of the curve right to the peak illustrates a high informativity of the analyzed region for the earliest evolutionary events, the part left to the peak, covering a large part of the analyzed timeline, indicates the accumulation of uninformative mutations^33, 34^. Therefore, the 3′ETS + NTS region is a suitable marker in shallower phylogenetic scale. Its application would not be appropriate in suprageneric level studies, as it would not reflect evolutionary history of higher taxonomic groups and could lead to erroneous conclusions. Another disadvantage resulting from applying in phylogenetic study a nucleotide fragment highly variable in length and sequence is a problem with alignment construction. However, identification of repeated motifs in NTS and the conservative TIS sites greatly facilitates aligning this extremely variable fragment, and in our opinion indeed is essential for the correct data analysis.

Arrangement of repeats distinguished in the NTS should best illustrate the evolutionary history of this region formed by multiplication or deletions of entire sequence fragments. Point mutations in type of indels or nucleotide substitutions happening simultaneously with motif multiplication obliterate their picture to a large extent. Therefore, assignment of best possible scheme of repeats is challenging and largely depending on available set of data. Having at disposal only one nucleotide sequence one would distinguish another set of repeats than while having set of sequences for several closely related species. Also, depending on the algorithm used for the designation of motifs, different set of repeats might be obtained. In this study methodology assumed the constant length of particular repeats, differing only in SNPs. In other studies^31, 35, 36^, authors decided to take into account the differences in the length of motifs. Due to different methodology, sometimes including searching for sequence motifs bye eye^31, 35^, often the results obtained in different studies are inconsistent with each other. Only in case of clear and simple patterns of repeats we can get similar results using various methods. That is a case of *Zea mays*, where ten IRs 192 bp in length distinguished in our study correspond with ten repeats between 165 and 232 nt in length described by McMullen *et al*.^35^. Also, the results of analysis of IGS from *Brachypodium distachyon* has a common ground with the results obtained by Borowska-Zuchowska *et al*.^37^ as described by them repeat REP corresponds with our B1 inter-repeat. The literature data on *Oryza sativa* demonstrated variation in IGS structure depending on the analyzed genotype, although the general scheme of alternating long and short repeat^38^ is similar to the pattern of repeats in IGS from rice analyzed in our study. In turn, the organization of IR region described by us for *Setaria italica* as an arrangement of two IRs repeated tandemly in five copies, doesn’t correspond to three regions of short subrepeats and one long repeat distinguished in IGS from *S*. *italica* by Fukunaga *et al*.^31^ who analyzed set of 40 accessions of landraces within two subspecies.

Our analysis of IGS structure within the genus of *Stipa* demonstrated its great applicability in phylogenetic studies at the generic level. However, the comparison of IGSs within the family of Poaceae leads to the conclusion that this region is highly divergent between the genera. Each of the analyzed genera was characterized by an individual pattern of inter-repeats and finding a common sequence motif or evolution scheme between the genera was impossible. Therefore, the IGS region is not an appropriate phylogenetic marker for studies at the supra-generic level in Poaceae.

The species included in the study also differed significantly regarding the presence of regions rich in AT sites. These regions are of special concern because they putatively contain protein binding sites involved in regulation of transcription and are associated with a proximity of gene promoter^8, 39, 40^. The presence of AT-rich sequence adjacent to TIS sites was reported, for example for *Arabidopsis thaliana* ^41^, *Olea europea* ^42^ *Haplopappus gracilis* ^39^, *Fagus sylvatica*, *Quercus suber* ^43^ and *Brassica oleracea* ^32^. Within the analyzed species representing Poaceae, the presence of AT-rich region upstream TIS was not a rule. Only in *Sorghum bicolor* three out of four short AT-rich regions, overlapping with fragments of SB1 repeat, preceded putative TIS sites. In *Stipa* spp. and *Setaria italica* a fragment of IGS representing a high rate of AT-sites was found, but closer to 25 S RNA gene, near the 5′ end of the NTS. In turn, the IGS from *Zea mays* contained four short AT-rich regions localized within the copies of IR Z1 but none of them was adjacent to TIS. The lack of IGS regions with a high share of AT pairs in *Oryza sativa* and *Brachypodium distachyon* completes the picture of sequence diversity within IGS.

The varied pattern of AT-rich sites distribution within the IGS may correspond with assumptions of some authors which discuss the significance of repeated elements in a control of nrRNA transcription. On the example of wheat, it was demonstrated that nrRNA genes located on the chromosomes with less subrepeats within the IGSs appeared relatively inactive^44^. Molecular studies showed that the subrepeat sequences in IGS attract similar proteins as sequences surrounding TIS and presumably play a role of promoter enhancers^8, 45, 46^. It is supposed that higher order structure formed by repeated motifs rather than a defined sequence plays a key role in these interactions^8, 32, 46^. In general, the IGS sequence is GC-rich^32^, however in the family of Poaceae the content of GC pairs is varied, and unless in *Oryza sativa* indeed it is very high and exceeds 70%, in *Sorghum bicolor* is less than 54%. Also in *Fagus sylvatica* and *Quercus suber* GC-content was at moderate level reaching 52% and 57% respectively. Characteristic for the IGS is the presence of CpG, CpCpG or CpNpG motifs, which are prone to methylation^8, 32, 47^. This one of the most important epigenetic modifications leads to gene silencing and the level of sequence methylation in the promoter region is strongly associated with the regulation of transcription of nuclear nrRNA genes^48^. It was shown that in species from Cucurbitaceae, equipped with a large number of ribosomal DNA, at least 70% of the repeats were completely methylated and therefore excluded from transcription^8^. The distribution of methylable sites along the IGS sequence is another feature of this spacer greatly diverse between the species^40^. For example, in *Punica granatum* over 100 methylable sites were detected, mainly in the subrepeat region and fewer within unique regions^40^. In turn, the IGS from *F*. *sylvatica* and *Q*. *suber* contained CpG island exclusively within the 5′ETS region^32^. Within the analyzed representatives of Poaceae there was no specific pattern of CpG sites distribution within the IGS in relation to its functional elements. In all the examined species, they were abundant and rather evenly distributed.

In conclusion, the present study shows that the IGS region, and especially its part adjacent to 26 S nrDNA, is a molecular marker giving a real chance for a phylogeny reconstruction of *Stipa*. The region seems to be the most phylogenetically informative for *Stipa* from all the chloroplast and nuclear markers tested so far. Moreover, as a nuclear spacer it enables the study of hybridization and introgression phenomena occurring in *Stipa*. Comparative analysis of nrDNA repeat units from *Stipa* to other representatives of Poaceae showed that their structure does not deviate from the general scheme. However, the rate of evolution within the inter-repeats region is extremely high and therefore it predestines the region for phylogenetic analyses of *Stipa* at the interspecific level in the genus.

## Materials and Methods

### Plant material and DNA extraction

All the examined *Stipa spp*. specimens were collected during field research in the years 2011–2014 (see Supplementary Table S3). Total genomic DNA was extracted from desiccated leaf tissue using ZR Plant/Seed DNA MiniPrep^TM^ kit (Zymo Research Corp., USA) and Genomic Mini AX Plant Spin (A&A Biotechnology, Poland) following the manufacturers recommendations. DNA quality was assessed by the 1% agarose gel electrophoresis and quantity was estimated with the use of the Qubit fluorometer system and the Quant-IT ds-DNA BR Assay kit (Invitrogen, USA).4.

### DNA library preparation and sequencing

A genomic library for MiSeq sequencing (for *S*. *lipskyi*, *S*. *narynica* & *S*. *orientalis*) was developed with the use of the Nextera XT Kit (Illumina, San Diego, CA, USA). DNA in the amount of 1 ng was used in the procedure described in the Nextera XT protocol. Constructed libraries were sequenced using the Miseq. 600v3 sequencing kit (Illumina, San Diego, CA, USA) that enable to obtain of 2 × 300-bp pair-end reads.

Three other species (*S*. *caucasica*, *S*. *magnifica*, *S*. *pennata*) were sequenced using the Illumina HiSeq. 2000 platform (Illumina, San Diego, CA, USA). A 350-bp paired-end library was constructed using Truseq DNA Nano kit and sequenced at Macrogene (Korea).

### NGS sequencing

All the obtained reads were trimmed and parts with low quality (Q below 5) or containing N’s were excluded. Cleaned reads were assembled de novo using Velvet plugin in Geneious 7.0 (Biomatters, New Zealand). The contigs contained nrRNA genes were identified and were further expanded by mapping cleaned reads with 25 iterations using Geneious 7.0 with custom settings (70 bp overlap with 99% identity). The sequences obtained after iterations were assembled de novo using Geneious built-in assembler with high sensitivity settings.

Annotations were performed using Geneious software based on BLAST tool and NCBI resources^49^. Complete IGS sequences of six *Stipa* species were explored to discover the degree of conservation between different functional units. Sequences were aligned with Muscle genome alignment within the Geneious environment and corrected manually. The nrDNA sequences of *Brachypodium distachyon*, *Oryza sativa*, *Setaria italica*, *Sorghum bicolor* and *Zea mays* downloaded from the NCBI database (Supplementary Table S4) were not included into the alignment due to the vast variability in IGS region which made an accurate sequence alignment impossible. Six complete nrDNA (ribosomal DNA) sequences were deposited in the NCBI (Supplementary Table S3).

### Sanger sequencing

PCR fragments for sequencing with Sanger method were amplified using TFL Epicentre polymerase and the primer combination: igsF: 5′-AGC CCC ACG TCG CAC GGA TTC GTC C-3′ with igsR: 5′-CCT CMC TTC AAC MGT TTC CRT GGG-3′. PCR experiments included an initial 95 °C denaturation followed by 35 cycles of 45 s at 95 °C, 50 s at 54-57 °C, 1 min 20 s at 72 °C, with a final 7-min extension at 72 °C. The amplification products were visualized on 2% agarose gel with GelView (Invitrogen™, Carlsbad, CA, USA) staining. Purified PCR products were sequenced in both directions using ABI BigDye 3.1 Terminator Cycle Kit (Applied Biosystems®, Foster City, CA, USA) with the same primers and then visualized using an ABI Prism 3130 Automated DNA Sequencer (Applied Biosystems®, Foster City, CA, USA). PCR recipe: 20 mM (NH_4_)SO_4_, 50 mM Tris-HCl (pH 9.0 at 25 °C), 1.5 mM MgCl_2_, 10 μg BSA, 0.2 mM of each dNTPs, 1.0 μM of each primer, 1U Taq polymerase (TFL Epicentre), 10 ng of the DNA, to 20 μl with dH_2_0.

### The analysis of IGS structure

For the discovery and comparative analysis of repetitive motifs in nucleotide sequences complete IGS regions were extracted from nrDNA units and submitted to the MEME Suite 4.11.2^50^. Discovery of sequence inter-repeats was done with the MEME tool^51^ and verified with MAST^52^. Six IGS sequences of *Stipa spp*. were submitted to the MEME environment as a set while each of the other sequences were analyzed individually. The limit for the minimum and maximum width of the repetitive motif was 20-200 bp for *Stipa spp*. and *Zea mays*, 15-250 bp for *Brachypodium distachyon*, and 25-250 bp for *Setaria italica*, *Sorghum bicolor* and *Oryza sativa*. The most suitable length of predicted inter-repeats was established basing on their E-values.

The prediction of putative transcription initiation sites (TIS) and putative transcription termination sites (TTS) was based on the comparative analysis of sequences with literature data concerning different plant species^8, 29, 32, 37, 43, 46^. AT-rich regions and CpG islands were identified using the Geneious software. The Geneious environment was also used to perform dot plot analysis.

### Phylogenetic informativeness

Phylogenetic informativeness (PI) was estimated using PhyDesign server^53, 54^. PI profiles were plotted with reference to an uncalibrated tree. The tree used to overlay the historic changes in substitution rates was obtained with Maximum Likelihood method using MEGA v.6^55^ and ultrametrisized using PATHd8^56^. To obtain relative ages for the clades, the root of the tree was set at an evolutionary time of 1.0 and tips at time of 0. The HyPhy program^57^ which is using empirical base frequencies and a time-reversible model of substitution was used to calculate PI of nucleotide data sets.

### The analysis of ITS and 3′ETS + NTS resolving power

MP analysis was done using MEGA v.6^55^ The MP tree was obtained using the Subtree-Pruning-Regrafting (SPR) algorithm with search level 1 and the number of initial trees equal to 10. The tree was tested with bootstrap method with the number of bootstrap replications at the level of 500.

BI analysis was done using MrBayes plugin in Geneious 7.0 (Biomatters, New Zealand) with the priors set according to the output of DNA model testing in MEGA v.6^55^. The parameters of the likelihood model applied for 3′ETS + NTS were adequate for general time reversible model with a gamma-shaped distribution of rates across sites (*GTR + Γ*), (*n*_*st*_ = 5). BI was estimated running four incrementally heated chains (MCMC algorithm) for 1,000,000 generations, sampling one out of every 200 generations of rando trees. The first 200,000 generations were discarded as “burn-in”. The remaining generations were used to construct The Bayesian consensus tree.

## Electronic supplementary material

Supplementary Information
