# Supplementary material for: Phylogenetic implications of nuclear rRNA IGS variation in Stipa L. (Poaceae)
Source: Sci Rep. 2017 Sep 14;7:11506. doi: 10.1038/s41598-017-11804-x (PMC5599551; doi:10.1038/s41598-017-11804-x)
Supplement: Supplementary file 1 — Supplementary Information [file 41598_2017_11804_MOESM1_ESM.doc]

Title: **Phylogenetic implications of nuclear rRNA IGS variation in *Stipa* L. (Poaceae)**

Authors: Katarzyna Krawczyk, Marcin Nobis, Arkadiusz Nowak, Monika Szczecińska, Jakub Sawicki

**Supplementary Information**

**Supplementary Table S1.** Comparison of putative TTS in different representatives of Poaceae. Position of the first TTS nucleotide for the putative TTS sequence with respect to the 3’ end of 25S-rDNA.

| Taxon | TTS sequence | TTS position |
| --- | --- | --- |
| *Stipa caucasica* | CCCCTTCTTCTC | 61 |
| *S. lipskyi* | CCCCTTCTTCTC | 61 |
| *S. narynica* | CCCTTTTTCTC | 62 |
| *S. magnifica* | CCCCTTTTTCTC | 68 |
| *S. orientalis* | CCCCACCTTTTTCTC | 64 |
| *S. pennata* | CCCCCCCGTTTTCTC | 64 |
| *Brachypodium distachyon* | CTCCCCCACC | 1 |
|  | CTTTGTTCCGCC | 61 |
| *Oryza sativa* | CCCTCTCCCCC | 9 |
|  | TTCTCCCCCCCCCTCCC | 34 |
| *Setaria italica* | CCCTCCACCCCC | 1 |
|  | CCCGCCAC | 34 |
| *Sorghum bicolor* | CCTCC | 1 |
|  | CCTCGC | 35 |
| *Zea mays* | CCCACCCT | 1 |
|  | TCTTCC | 58 |

**Supplementary Table S2.** Comparison of putative TIS in different representatives of Poaceae. Position of the first TIS nucleotide for the putative TIS sequence with respect to the 5' end of the 18S-DNA. The positions for *Stipa spp*. are given for the sequence alignment, numbers in brackets - for the species

| Taxon | TIS sequence | TIS position |
| --- | --- | --- |
| *Stipa* spp*.* | TATAGTGTGGGG | 686 (677-684) |
|  | TATATTGTGGGG | 991 (982-989) |
| *Brachypodium distachyon* | TATAGTAGGGG | 879 |
|  | TATAGTAGGGGG | 1182 |
|  | TATAGTAGGGTTGG | 1605 |
|  | TATAGTAGGGGG | 1740 |
| *Oryza sativa* | TATAGCAGGGTGG | 1103 |
|  | TATAGTAGGGGTG | 1357 |
|  | TATAGTAGGGGGG | 1611 |
| *Setaria italica* | TATTCGGGGTGT | 779 |
|  | TATAGTAGGGGG | 869 |
| *Sorghum bicolor* | TATAGGGGAGAGGG | 645 |
|  | TATAGGGGAGAGGG | 1084 |
|  | TATAGGGGAGAGGG | 1613 |
| *Zea mays* | TATAGTAGGGGG | 830 |

**Supplementary Table S3**. Specimens used in the study

| Taxon | Country | Voucher | GB accession number |
| --- | --- | --- | --- |
| *Achnatherum chingii* (Hitchc.) Keng | China | Quighai, Twelve winding slopes, 28 July 2010, B. Paszko (KRA); A/4/2 | KY826208 |
| *Stipa arabica* Trin. & Rupr. | Kyrgyzstan | Toktogul, 5 July 2013, M. Nobis & A. Nowak (KRA); S/61/4 | KY826209 |
| *S. baicalensis* Roshev. | Russia | Khakasia Resp., Shirinskii distr., 6 km of Irbitsk, 21 July 2008, P.D. Gudkova (KRA); S/3/1 | KY826210 |
| *S. bungeana* Trin. ex Bunge | Kyrgyzstan | Tian-San Mts, NW part of Issyk-kul Lake, 11 Jun 2013, M. Nobis, A. Nowak (KRA); S/28/1 | MF405147 |
| *S. capillacea* Keng | Bhutan | Thimphu distr., Thimphu hospital, alt. 3300 m, 22 July 1989 J.R.I. Wood 7033 (E); S/64/1 | KY826211 |
| *S. capillata* L. | Poland | Wola Zagojska village, 3 June 2012, M. Nobis (KRA); S/4/1 | KY826212 |
| *S. caucasica* Schmalh. | Kazakhstan | 150 km W of Almaty, 18 May 2014, M. Nobis, P. Gudkova (KRA); S/53/1 | KY826229 |
| *S. dasyphylla* (Lindem.) Trautv. | Slovakia | 14 June 2012, R. Piwowarczyk (KRA); S/81/1 | KY826213 |
| *S. drobovii* (Tzvelev) Czerep. | Tajikistan | Zeravshan Mts, Mogien settl., 25 May 2015, M. Nobis (KRA); S/11/5 | MF405148 |
| *S. eriocaulis* Borb. | Czech Republic | Mikulov, 5 May 2012, M. Nobis & A. Nobis (KRA); S/80/1 | KY826214 |
| *S. glareosa* P.A.Smirn. | Tajikistan | Murgab, 4 Jul 2008, M. Nobis, (KRA); S/49/1 | MF405149 |
| *S. gracilis* Roshev. | Kyrgyzstan | Alai Mts, Abshzr-Ata, 1 Jul 2015, M. Nobis (KRA); S/65/1 | MF405150 |
| *S. grandis* P.A.Smirn | Russia | 6 km NW of Inzagatui village, 1 August 2014, M. Nobis (KRA); S/1/3 | KY826215 |
| *S. holosericea* Trin. & Rupr. | Greece | Parnas Mts, NNE of Delf, 1 May 2010, R. Piwowarczyk (KRA); S/38/2 | KY826230 |
| *S. karataviensis* Roshev. | Kazakhstan | Khantau Mts, Khantau settl., 14 May 2014, A.L. Ebel (KRA); S/66/1 | MF405151 |
| *S. kirghisorum* P.A.Smirn. | Tajikistan | Sarytag, 15 Jun 2007, M. Nobis, A. Nowak (KRA); S/48/2 | MF405152 |
| *S. krylovii* Roshev. | Mongolia | 50 km SW of Gurvanzagal, 15 August 2011, Safronova et al. (KRA); S/2/1 | KY826216 |
| *S. lessingiana* Trin. & Rupr. | Kyrgyzstan | Tian-Shan Mts, betweene Chat and Alcha settl., 1170 m, 10 May 2011, M. Nobis (KRA); S/20/1 | KY826217 |
| *S. lingua* Junge | Tajikistan | Pamir Mts, Khorog, 1 Jul 2008, M. Nobis (KRA); S/32/1 | MF405153 |
| *S. lipskyi* Roshev. | Tajikistan | Zeravshan Mts, Vashan River valley, 1680 m, 21 June 2010, M. Nobis (KRA); S/17/4 | KY826233 |
| *S. macroglossa* P.A.Smirn. | Tajikistan | Zeravshan Mts, Mogien, 25 May 2015, M. Nobis (KRA); S/44/1 | MF405154 |
| *S. magnifica* A.Junge | Kyrgyzstan | Tian-Shan Mts, Tashkumyr, 650 m, 11 May 2011, M. Nobis, A. Nowak (KRA); S/21/1 | KY826234 |
| *S. narynica* M.Nobis | Kyrgyzstan | E of Tash Kumyr, 11 May 2011, M. Nobis, A. Nowak (KRA); S/74/1 | KY826235 |
| *S. offneri* Breistr. | Spain | Montserrat, May 2009, R. Piwowarczyk (KRA); S/8/1 | KY826218 |
| *S. orientalis* Trin. | Tajikistan | Zeravshan Mts, near Iskanderkul Lake, 2300 m, 15 June 2011, M. Nobis (KRA); S/24/2 | KY826231 |
| *S. pennata* L. | Poland | Skarpy Ślesińskie near Slesin, May 2015, E. Klichowska (KRA); S/42/16 | KY826232 |
| *S. przewalskyi* Roshev. | China | Qinghai, 1 Aug 2010, B. Paszko (KRA); S/18/1 | MF405155 |
| *S. purpurea* Griseb. | China | Qinghai prov., NE of Dashui Bridge, 3617 m, 24 July 2010, B. Paszko (KRA); S/5/2 | KY826219 |
| *S. regeliana* Hack. | Kyrgyzstan | S of Barksoon, 9 July 2015, M. Nobis, A. Nowak (KRA); S/37/2 | KY826220 |
| *S. richteriana* Kar. & Kir. | Kazakhstan | Balkhash, 22 May 2014, M. Nobis (KRA); S/70/1 | KY826221 |
| *S. sareptana* A.Beck. | Kyrgyzstan | 7.5 km SE of Dzhel-Aryk, 1345 m, 15 May 2011, M. Nobis (KRA); S/25/1 | KY826222 |
| *S. subsessiliflora* (Rupr.) Roshev. | Tajikistan | near Karakol Lake, 23 August 2013, A. Nowak (KRA); S/6/1 | KY826223 |
| *S. tianschanica* Roshev. | Tajikistan | Pamir, Alichur, 24 August 2013, A. Nowak (KRA); S/26/4 | KY826224 |
| *S. tirsa* Stev. | Armenia | Antarut settl., 8 July 2015, R. Piwowarczyk (KRA); S/84/1 | KY826225 |
| *S. trichoides* P.A.Smirn. | Tajikistan | Margeb, 8 June 2011, M. Nobis (KRA); S/46/1 | KY826226 |
| *S. turkestanica* Hack. | Tajikistan | Shivoz, 1 July 2008, M. Nobis (KRA); S/47/1 | KY826227 |
| *S. zalesskii* Wilensky | Kyrgyzstan | 12 km NW of Sassumyr, 7 July 2015, M. Nobis, A. Nowak (KRA); S/83/1 | KY826228 |

Supplementary Table S4. GenBank accession numbers for DNA sequence data

| Taxon | GB accession number | Chromosome | Bases nrDNA |
| --- | --- | --- | --- |
| *Brachypodium distachyon* | NC_016131.2 | 5 | 8 309 – 16 386 |
| *Oryza sativa* | AP_008208.2 | 2 | 29 600 467 – 29 608 389 |
| *Setaria italica* | NC_028456.1 | 7 | 36 184 – 43 962 |
| *Sorghum bicolor* | CM_000764.2 | 5 | 35 940 545 – 35 949 233 |
| *Zea mays* | NW_007617766 | 10 | 88 076 770 – 88 085 564 |

**
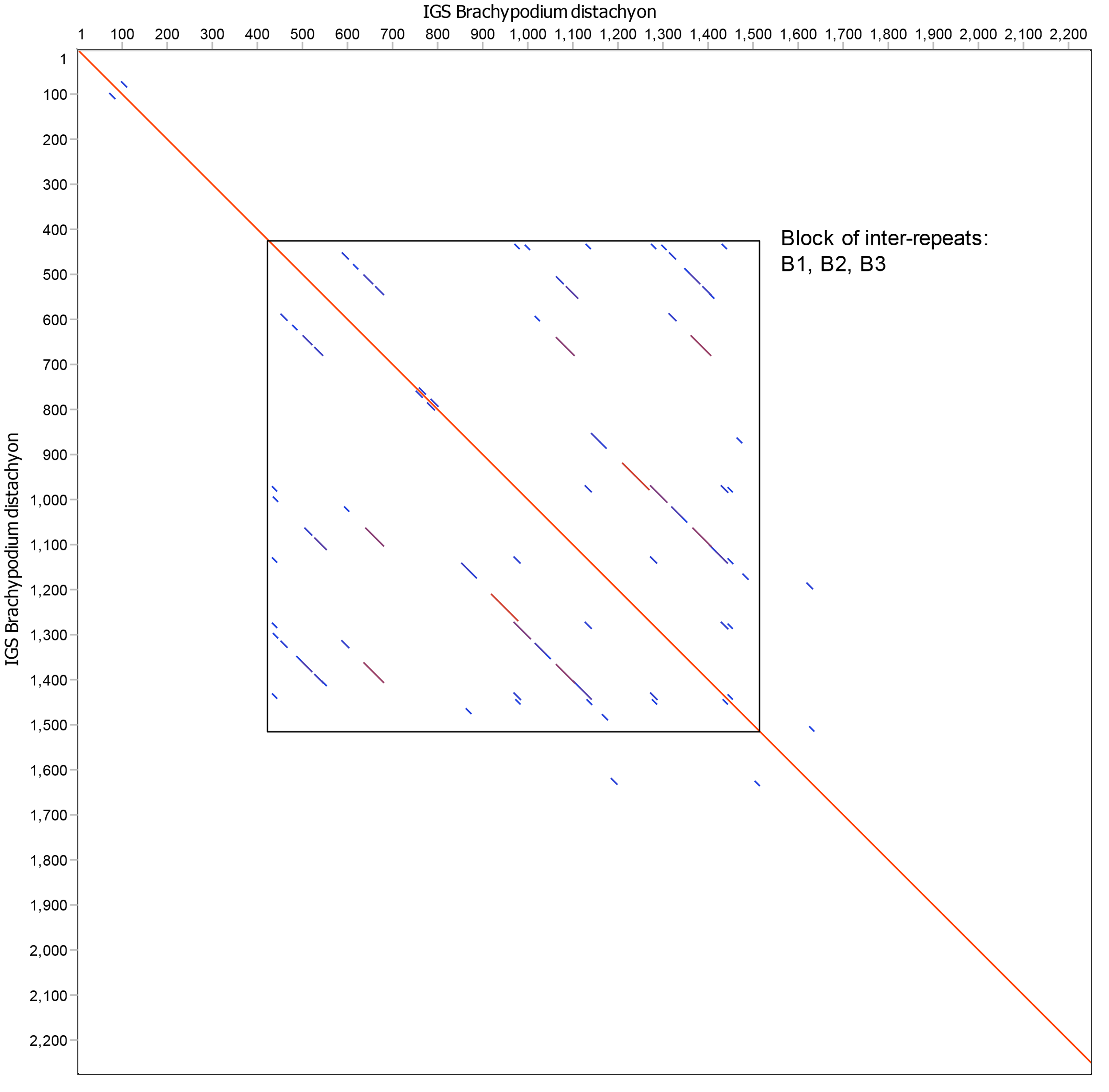
**

**Supplementary Figure S5.** Dot matrix plot of intergenic spacers. Self-comparison of *Brachypodium distachyon* IGS. The range of frame comprising block of inter-repeats corresponds with the length of inter-repeats in Figure 5, Figure 6 and in Table 2.


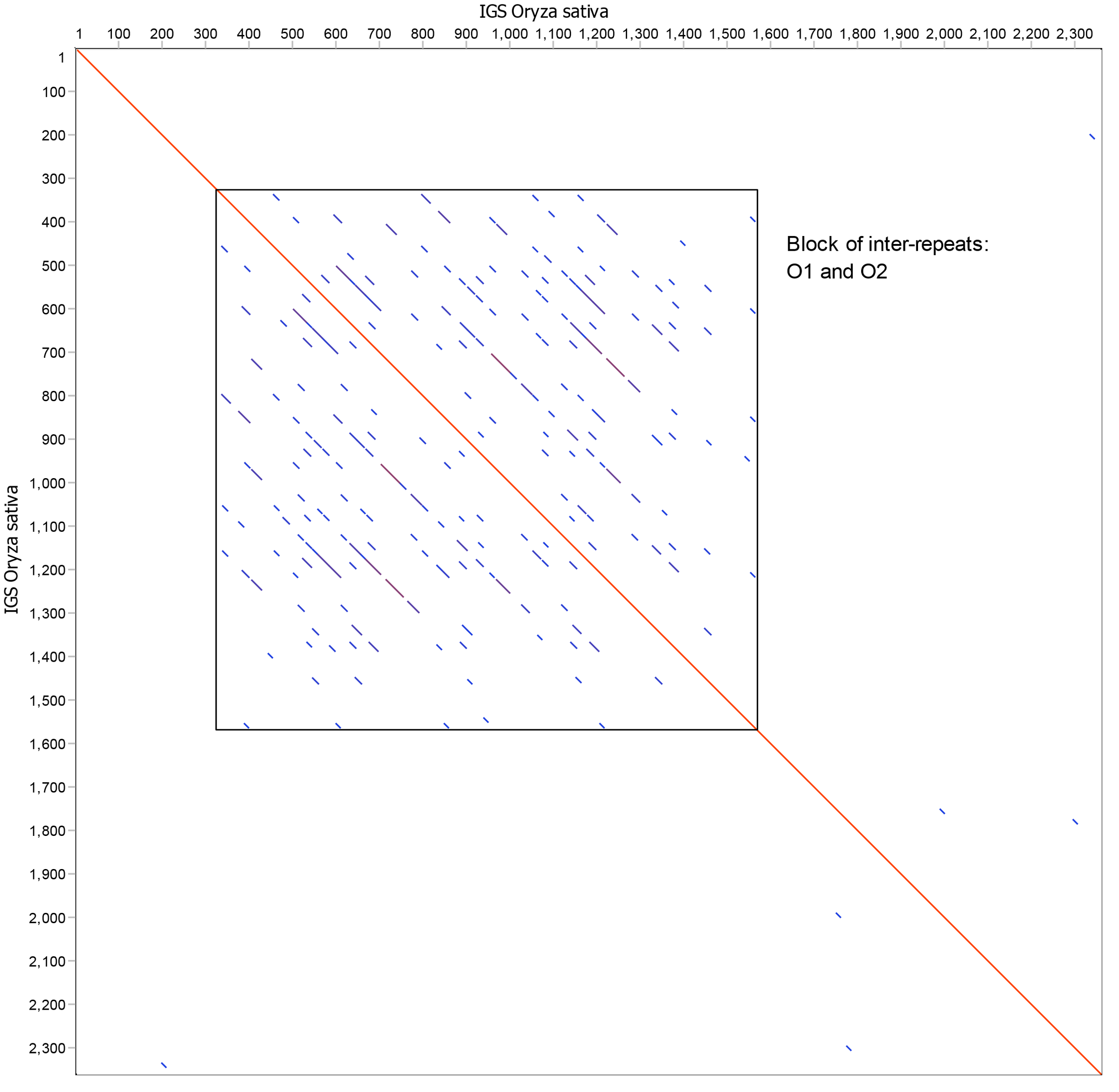


**Supplementary Figure S6.** Dot matrix plot of intergenic spacers. Self-comparison of *Oryza sativa* IGS. The range of frame comprising block of inter-repeats corresponds with the length of inter-repeats in Figure 5, Figure 6 and in Table 2.


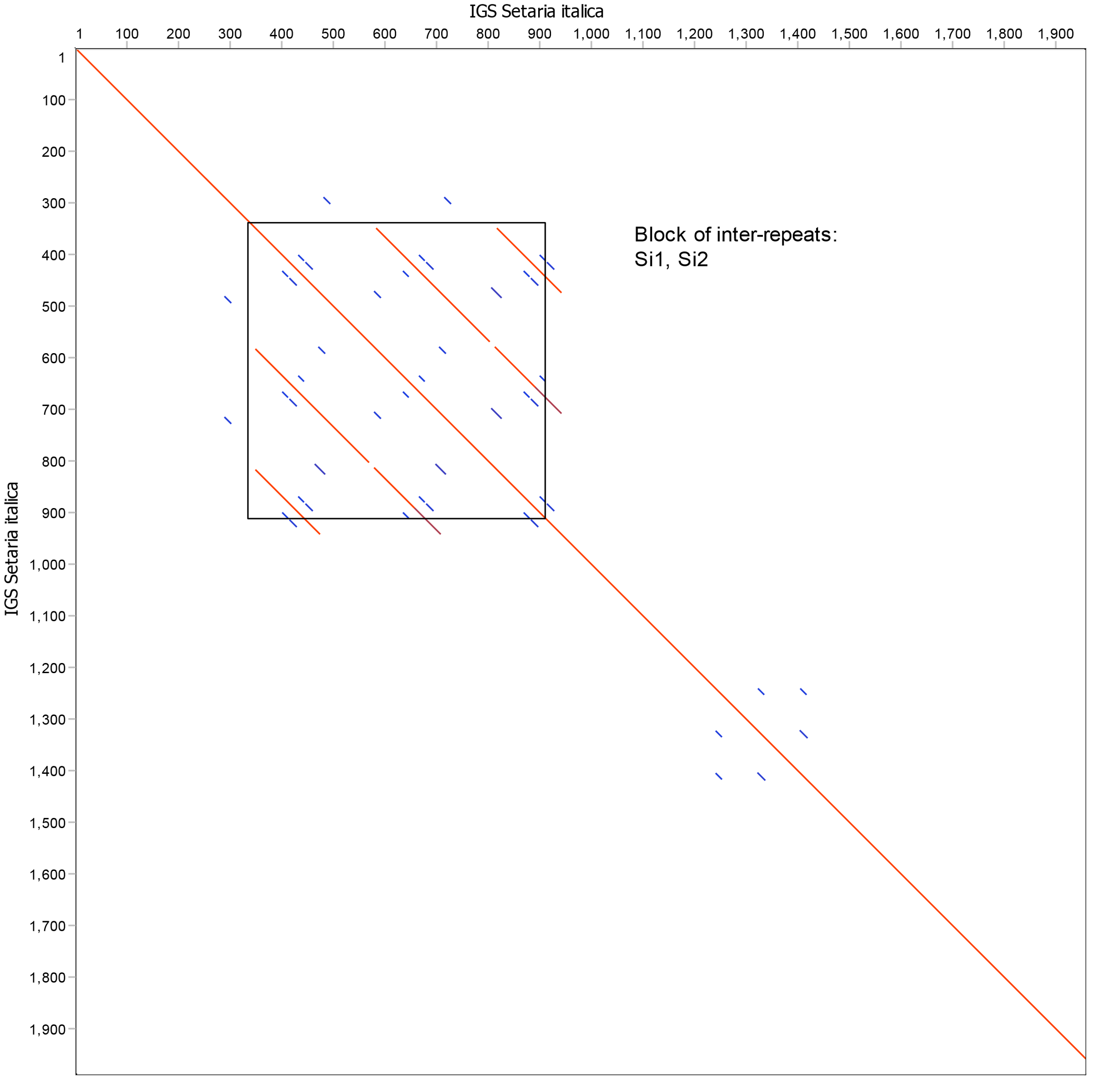


**Supplementary Figure S7.** Dot matrix plot of intergenic spacers. Self-comparison of *Setaria italica* IGS. The range of frame comprising block of inter-repeats corresponds with the length of inter-repeats in Figure 5, Figure 6 and in Table 2.


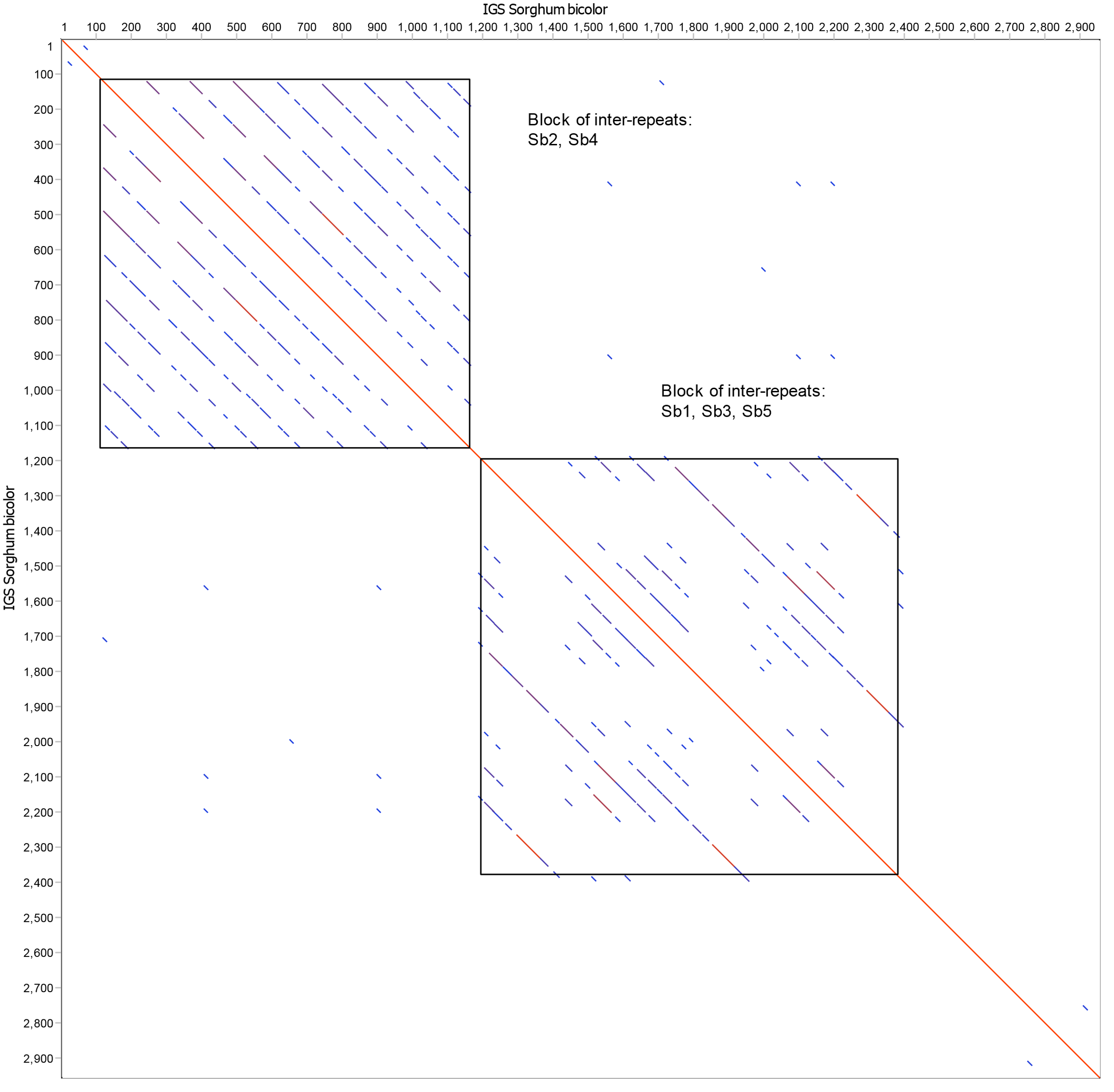


**Supplementary Figure S8.** Dot matrix plot of intergenic spacers. Self-comparison of *Sorghum bicolor* IGS. The range of frame comprising block of inter-repeats corresponds with the length of inter-repeats in Figure 5, Figure 6 and in Table 2.


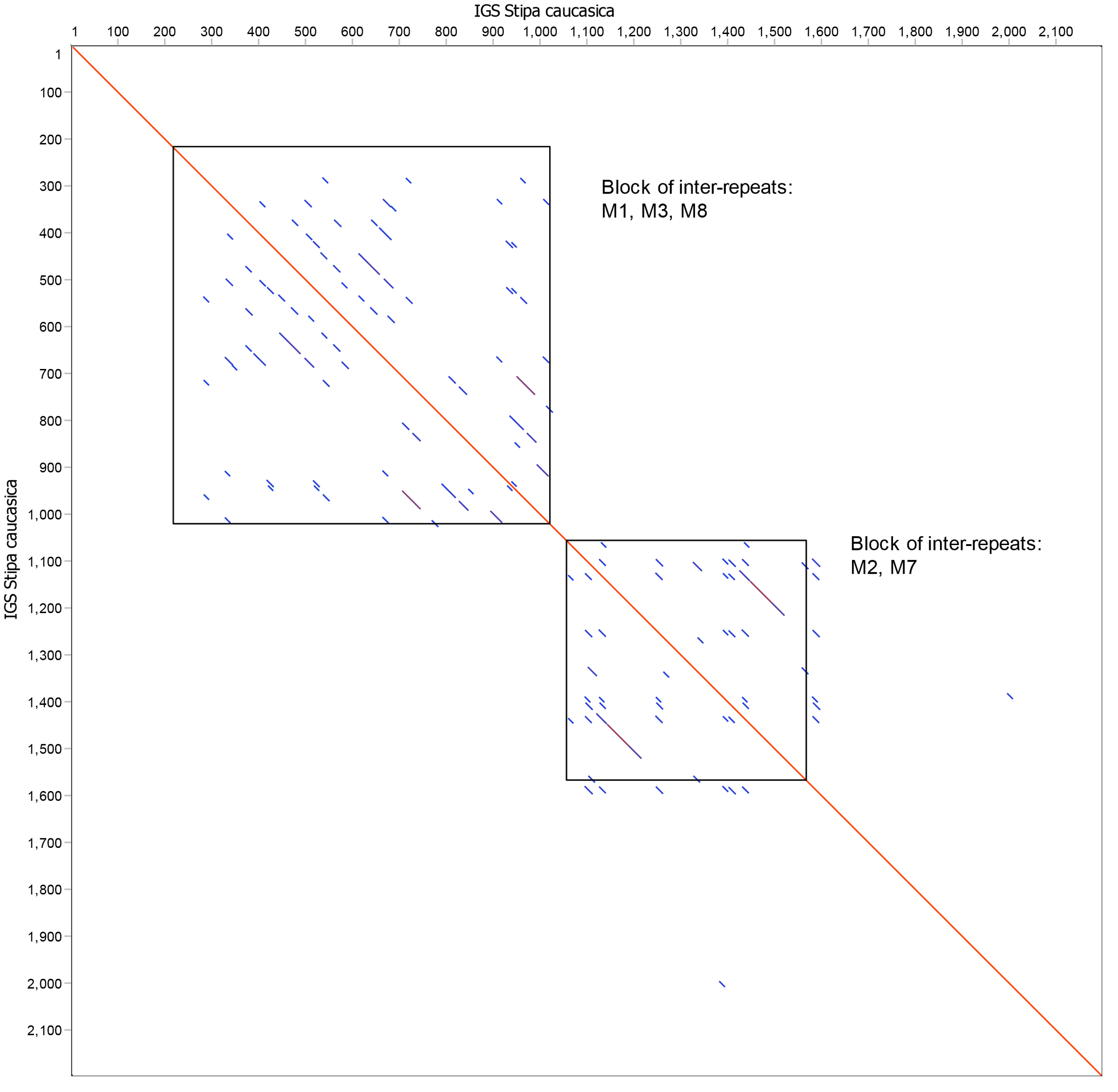


**Supplementary Figure S9.** Dot matrix plot of intergenic spacers. Self-comparison of *Stipa caucasica* IGS. The range of frame comprising block of inter-repeats corresponds with the length of inter-repeats in Figure 5, Figure 6 and in Table 2.


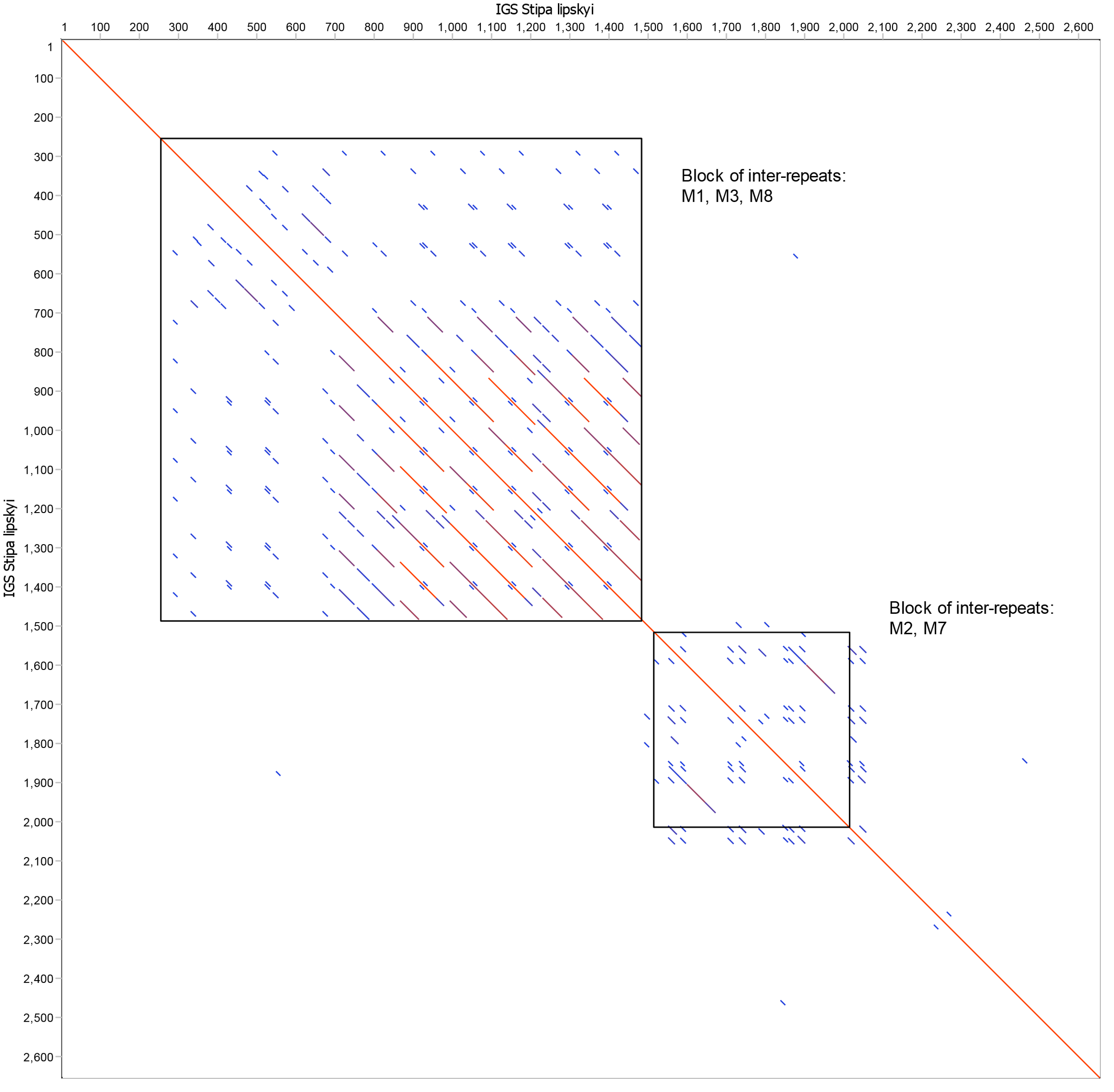


**Supplementary Figure S10.** Dot matrix plot of intergenic spacers. Self-comparison of *Stipa lipskyi* IGS. The range of frame comprising block of inter-repeats corresponds with the length of inter-repeats in Figure 5, Figure 6 and in Table 2.


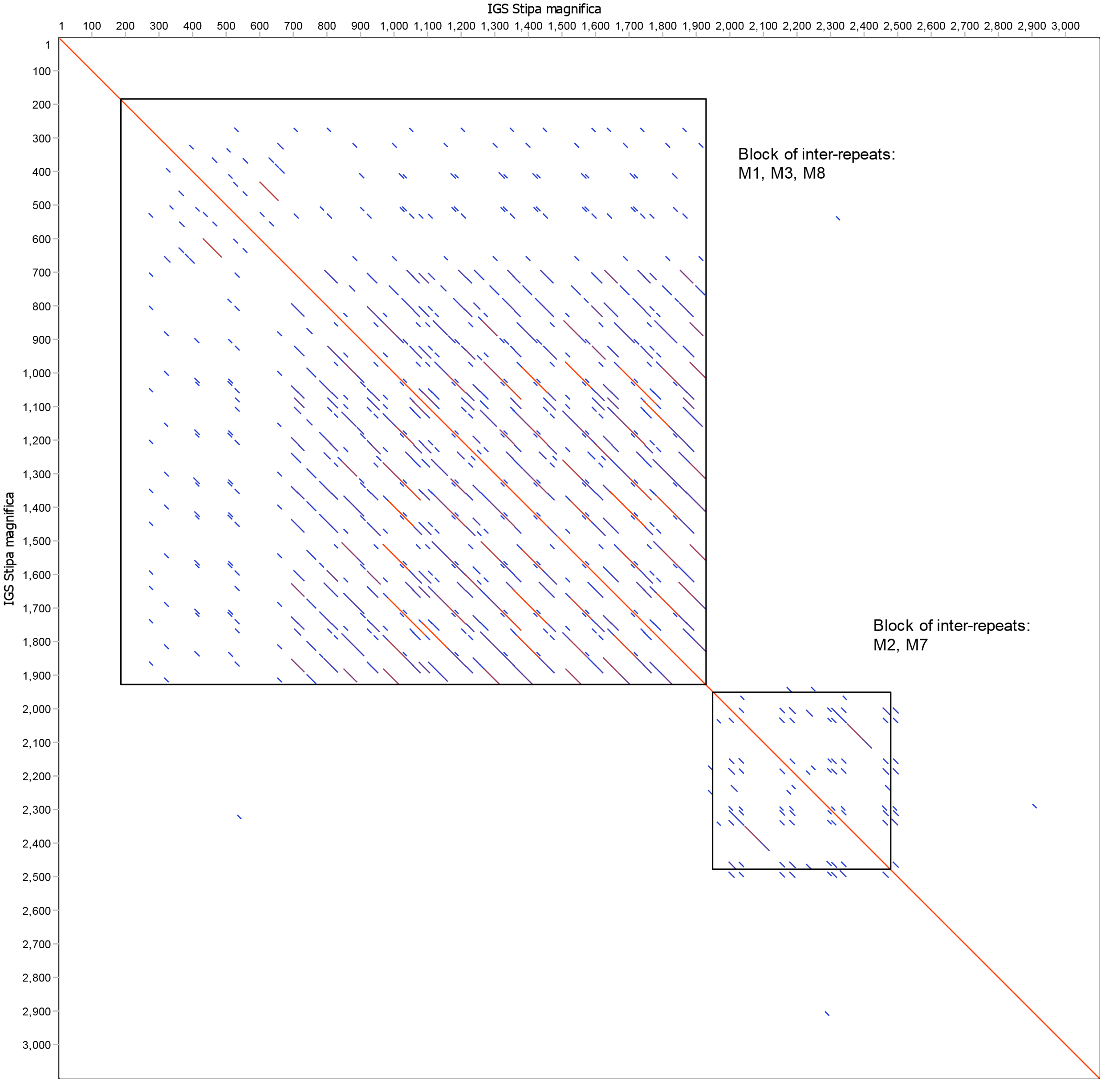


**Supplementary Figure S11.** Dot matrix plot of intergenic spacers. Self-comparison of *Stipa magnifica* IGS. The range of frame comprising block of inter-repeats corresponds with the length of inter-repeats in Figure 5, Figure 6 and in Table 2.


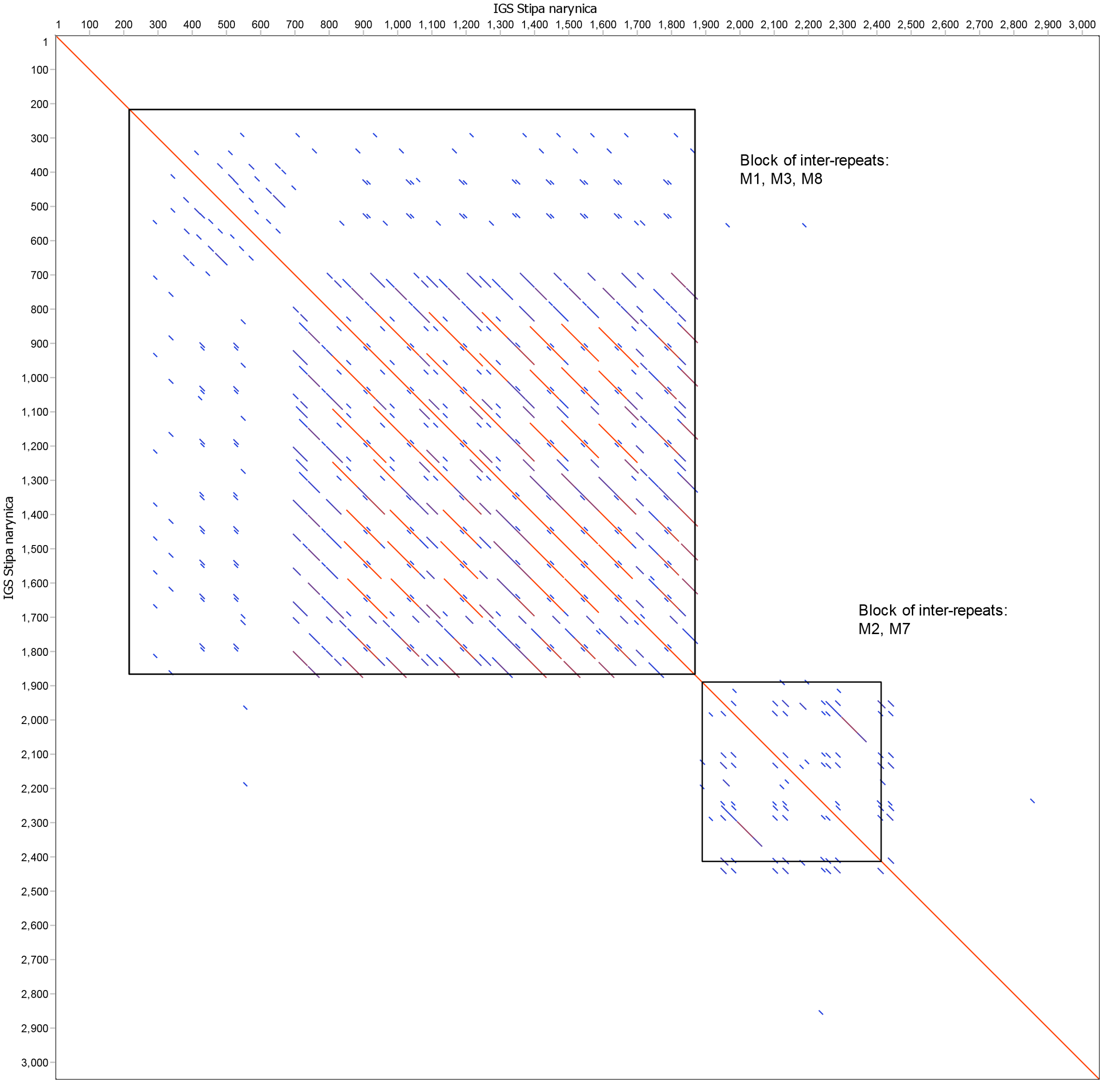


**Supplementary Figure S12.** Dot matrix plot of intergenic spacers. Self-comparison of *Stipa narynica* IGS. The range of frame comprising block of inter-repeats corresponds with the length of inter-repeats in Figure 5, Figure 6 and in Table 2.


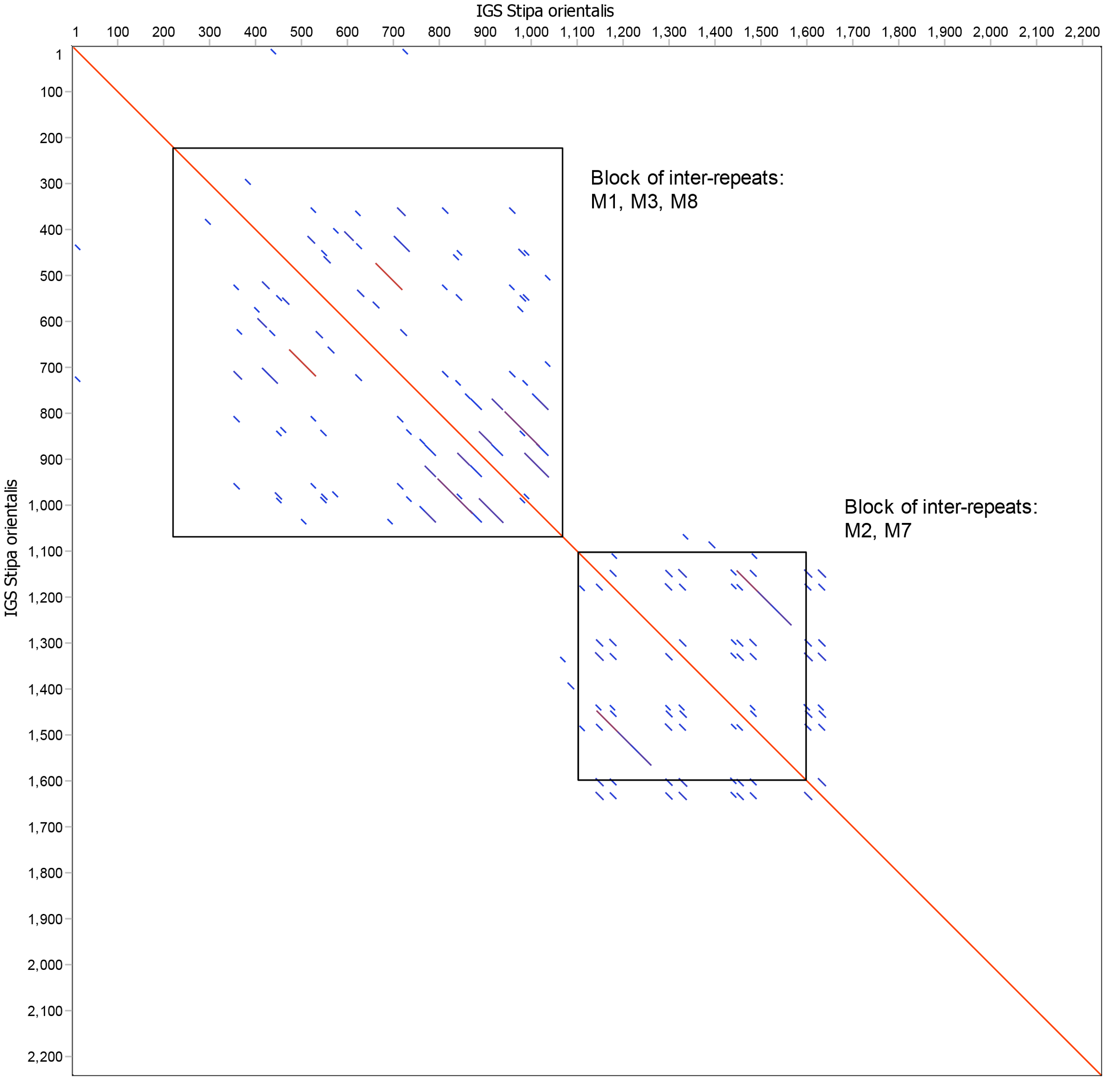


**Supplementary Figure S13.** Dot matrix plot of intergenic spacers. Self-comparison of *Stipa orientalis* IGS. The range of frame comprising block of inter-repeats corresponds with the length of inter-repeats in Figure 5, Figure 6 and in Table 2.


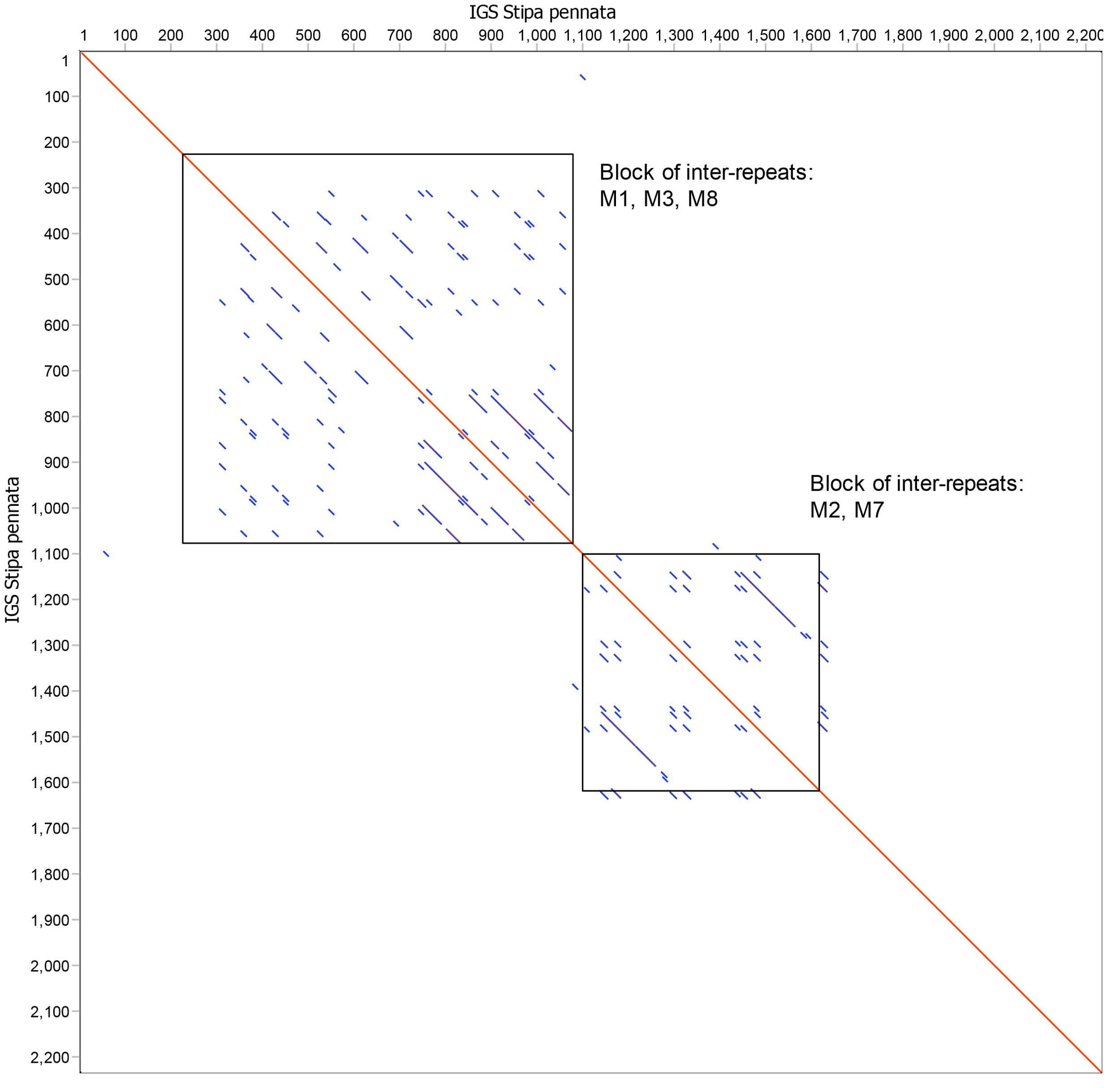


**Supplementary Figure S14.** Dot matrix plot of intergenic spacers. Self-comparison of *Stipa pennata* IGS. The range of frame comprising block of inter-repeats corresponds with the length of inter-repeats in Figure 5, Figure 6 and in Table 2.

**
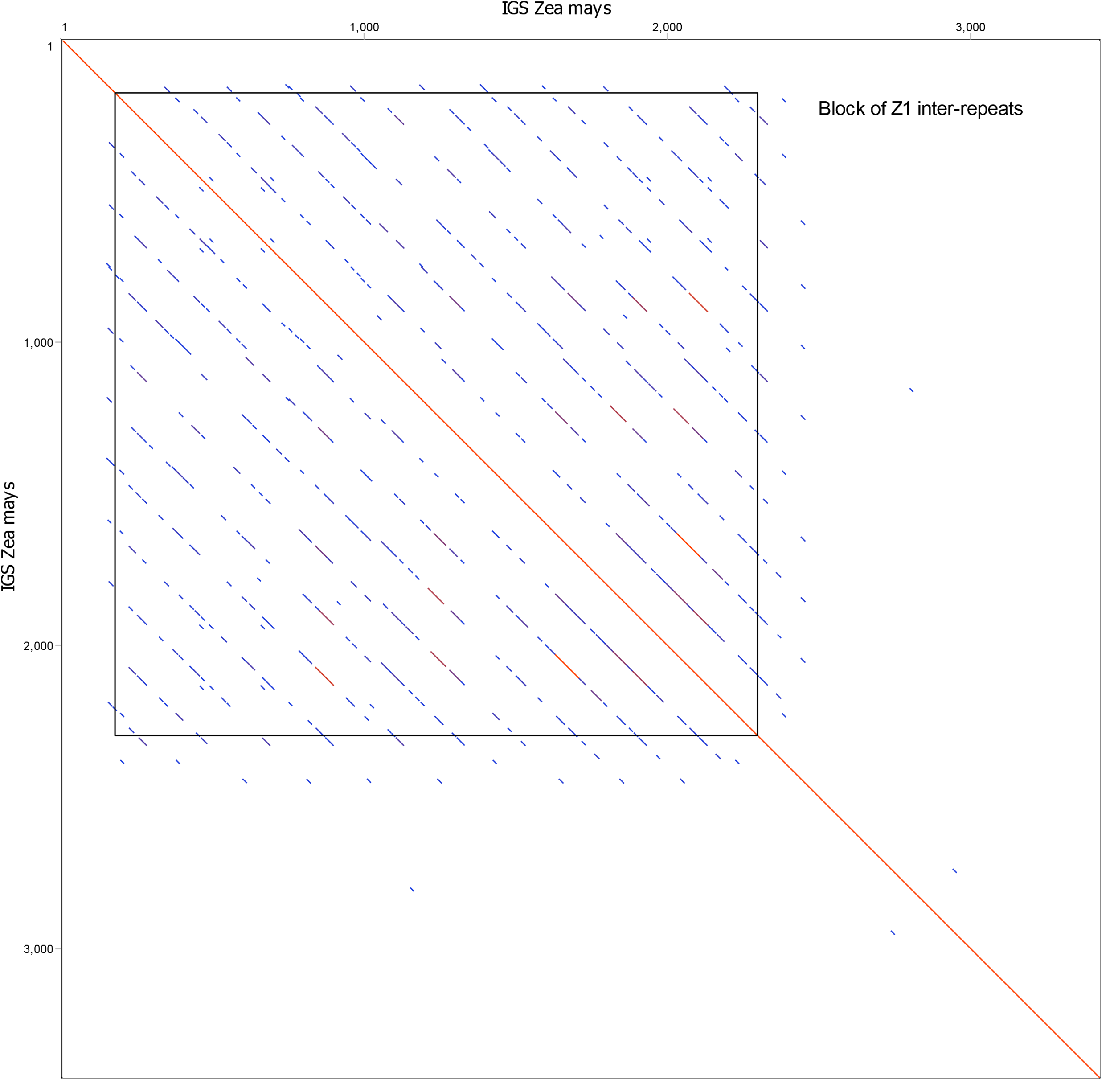
**

**Supplementary Figure S15.** Dot matrix plot of intergenic spacers. Self-comparison of *Zea mays* IGS. The range of frame comprising block of inter-repeats corresponds with the length of inter-repeats in Figure 5, Figure 6 and in Table 2.
